# Yellow protein co-opted to sustain obligate symbiosis in leaf beetles

**DOI:** 10.1038/s41467-026-76942-1

**Published:** 2026-09-03

**Authors:** Marleny García-Lozano, Christiane Emmerich, Christine Henzler, Iris Koch, Christa Lanz, Aftab Mahmood Ayas, Inès Pons, Anja Buttstedt, Katharina Hipp, Hassan Salem

**Affiliations:** 1 Mutualisms Research Group, Max Planck Institute for Biology, Tübingen, Germany; 2 Department of Molecular Microbiology, John Innes Centre, Norwich, UK; 3 The Sainsbury Laboratory, Norwich, UK; 4 Electron microscopy facility, Max Planck Institute for Biology, Tübingen, Germany; 5 Genome Centre, Max Planck Institute for Biology, Tübingen, Germany; 6 Institute for Evolution and Ecology, University of Tübingen, Tübingen, Germany

**Keywords:** Coevolution, Microbial ecology, Symbiosis, Non-model organisms

## Abstract

Yellow proteins are best known for their roles in pigmentation, behavior, and development across insects. Here, we uncover their striking evolutionary co-option for a wholly distinct function: sustaining a Paleocene-aged digestive symbiosis in tortoise beetles. We show that a female-specific Yellow forms the gelatinous spheres that encapsulate the bacterium *Stammera* during vertical transmission, allowing it to subsist extracellularly despite its drastically reduced genome (0.24 Mb) and limited metabolic capacity. *Yellow* expression is highly localized to symbiont-harboring glands in the ovaries, where the protein is assembled into a matrix and secreted during egg-laying. Functional knockdown of *yellow* disrupts sphere integrity and compromises symbiont viability under dry conditions, underscoring the protein’s embedding properties and protective role for *Stammera*. These findings reveal a novel function for an ancient gene family and demonstrate how tortoise beetles have repurposed Yellows to overcome the extreme metabolic constraints faced by their symbionts during extracellular transmission.

## Abstract (web summary)

Insects have evolved diverse strategies to protect their genome-reduced symbionts during vertical transmission. Here, authors show that tortoise beetles have co-opted a Yellow protein to form spheres that protect and transmit the symbiont *Stammera*.

## Introduction

First discovered over 80 years ago for their role in fruit fly pigmentation, Yellow proteins have since become a model for studying the genetic basis of form and function^1–7^. In one of the earliest demonstrations linking genes to complex behavior, mutations in *yellow* altered melanin-based coloration and disrupted male courtship success in *Drosophila melanogaster*^1^.

Yellow proteins span the insect phylogeny and are characterized by a six-bladed β-propeller fold^2,3,8,9^. Gene duplication and neofunctionalization have expanded the Yellow family into at least ten distinct subgroups, each with context-specific roles in development, immunity, and metabolism^5,8,10–14^. In honey bees (Hymenoptera), this expansion includes a specialized clade known as major royal jelly proteins (MRJPs); highly abundant in royal jelly, the nutritive secretion fed to larvae destined to become queens^12,15,16^. In flies (Diptera) and beetles (Coleoptera), Yellow proteins have been implicated in cuticular pigmentation^2,5–7,17^, chorion formation^18,19^, immune defense^8^, and stress responses^20^. Functions related to melanin synthesis and deposition have also been documented in wasps (Hymenoptera)^21^, butterflies (Lepidoptera)^22–26^, and grasshoppers (Orthoptera)^27^, underscoring the functional versatility and complexity of the Yellow protein family in insect adaptation.

**Fig. 1:**
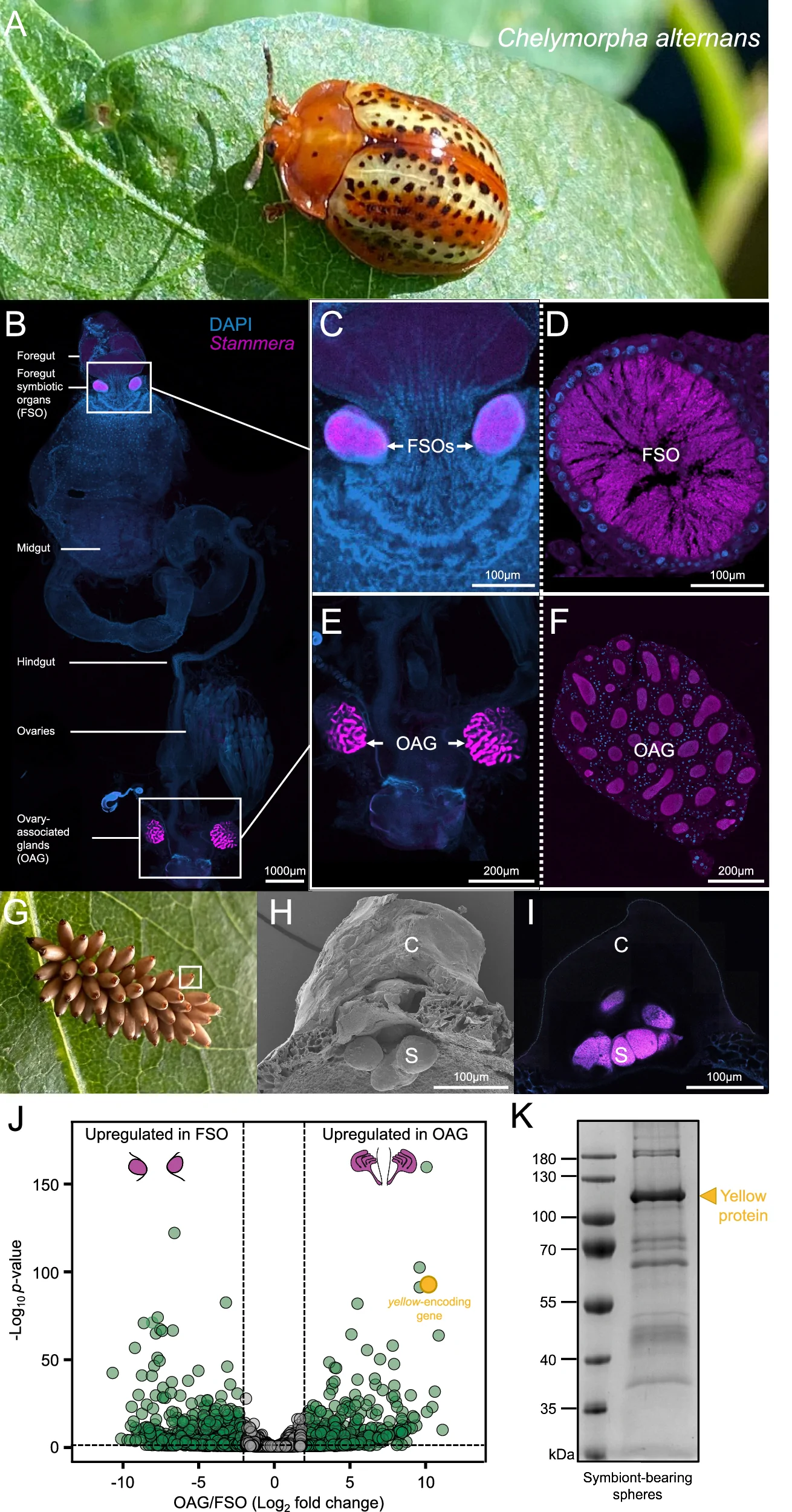
*Stammera*-bearing spheres are proteinaceous and are predominantly composed of Yellow. **A** Adult female *Chelymorpha alternans*. **B** Whole-mount fluorescence in situ hybridization (FISH) of an adult female’s digestive and reproductive systems, targeting *Stammera* (magenta: 16S rRNA). DAPI (blue) was used for DNA staining. **C**,**D** FISH of the foregut symbiotic organs. Whole-mount (**C**) and cross-section (**D**). **E**,**F** FISH of the ovary-associated glands. Whole-mount (**E**) and cross-section (**F**). **G** Eggs of *C. alternans*, each topped with a caplet (squared) at the anterior pole. **H** Scanning electron microscopy (SEM) image of an experimentally fractured egg caplet showing the symbiont-bearing spheres. **I** Cross-section FISH of an egg caplet, targeting *Stammera* (magenta: 16S rRNA) within symbiont-bearing spheres. The caplet outline has been manually drawn to define its edges. **J** Volcano plot showing differential gene expression between foregut symbiotic organs (*n* = 3 biological replicates) and ovary-associated glands (*n* = 3 biological replicates) analyzed via DESeq2 based on a negative binomial generalized linear model with a two-sided Wald test. P values were adjusted for multiple comparisons using the Benjamini-Hochberg false discovery rate (FDR) method. Genes significantly upregulated or downregulated in ovary-associated glands (log₂ fold change > 2, FDR-adjusted *p* < 0.05) are located in the upper right and left quadrants, respectively. The candidate gene is highlighted in yellow. The figures illustrating the foregut symbiotic organs and ovary-associated glands were created in Biorender. García-Lozano, M. (2026) https://BioRender.com/16nn3j4; García-Lozano, M. (2026) https://BioRender.com/sg2u5l8. **K** Sodium dodecyl-sulfate polyacrylamide gel electrophoresis (SDS-PAGE) of total proteins from symbiont-bearing spheres isolated from eggs; Yellow protein band is indicated. Abbreviations: C, caplet; S, sphere; FSO, foregut symbiotic organs; OAG, ovary-associated glands; kDa, kilodalton. Micrographs in Figs. 1B, F, H, I, k are from one individual and are shown for illustrative purposes. Source data are provided as a Source Data file.

Here, we extend this body of work by describing a novel role for Yellow proteins in insects: sustaining obligate symbiosis with beneficial microbes. Using tortoise beetles (Coleoptera: Chrysomelidae: Cassidinae) as a study system, we demonstrate the key role Yellow plays in embedding and propagating their obligate bacterial symbiont, *Candidatus* Stammera capleta (Fig. 1A)^28–35^.

Tortoise beetles maintain *Stammera* within specialized foregut symbiotic organs that facilitate folivory (Fig. 1B–D) and in ovary-associated glands that ensure the microbe’s vertical transmission^28–31,33,36,37^ (Fig. 1B, E, F). Despite possessing a highly reduced genome ( ~ 0.2–0.3 Mb), *Stammera* encodes and secretes plant cell wall-degrading enzymes, enabling the host to digest plant material enriched in recalcitrant polymers such as pectin and cellulose^28,29,31,32,34,35,37,38^.

During oviposition, females deposit egg caplets at the anterior pole of each egg^28,30^. Caplets are packaged with spherical secretions where *Stammera* is embedded extracellularly^28,30^. The *Stammera*-bearing spheres are ingested by the developing embryo (Fig. 1G–I), which initiates infection^28,30,33,36^. Experimental removal of the egg caplet disrupts the transmission of *Stammera*, yielding symbiont-free (aposymbiotic) insects^28,30^. These larvae exhibit a diminished digestive phenotype and low survivorship, demonstrating the obligately beneficial role *Stammera* plays for host development^28,30,36^.

Unlike other insect symbionts possessing drastically reduced genomes ( < 0.3 Mb), *Stammera* is transmitted extracellularly and must subsist for ~11 days within the egg caplet prior to its acquisition by the developing embryo^28,30,33^. Tortoise beetles are thought to sustain this extracellular transfer through the embedding properties of their egg-associated spheres^28,30^. While the ovary-associated glands have been shown to produce the spheres^28,30^, the composition of these secretions was yet to be characterized.

In this study, we demonstrate that the *Stammera*-bearing spheres are proteinaceous and are primarily composed of a Yellow protein. Specifically, we show that (i) a *yellow* gene is uniquely and highly expressed in the ovary-associated glands of adult females and is conserved across multiple tortoise beetle species; (ii) the corresponding Yellow protein forms a dense matrix, constituting the spheres where *Stammera* is embedded; and (iii) RNA interference (RNAi)-mediated knockdown of *yellow* disrupts sphere morphology and increases symbiont susceptibility to desiccation, highlighting the protein’s protective role during extracellular transmission. Collectively, our findings uncover a novel mechanism for symbiont encapsulation and propagation, predicated on the gelatinous properties of Yellow, and showcasing the remarkable functional versatility of this protein family in insect development.

## Results

### Tortoise beetles embed *Stammera* in Yellow during transmission

Obligate bacterial symbionts often experience severe population bottlenecks during vertical transmission, accelerating genome degeneration through the accumulation of deleterious mutations and the loss of genes involved in DNA replication, repair, and recombination^39^. As a result, symbionts become increasingly reliant on host support, especially during transmission when they may be exposed to harsh extracellular environments^40–43^. To mitigate these challenges, insect hosts leverage a variety of protective structures, including hydrocarbon coatings^44^, milk-like secretions^45^, jelly-like matrices^41^, and symbiont-bearing egg capsules^42,46^. In stinkbugs, a host-derived odorant-binding protein was recently identified as a key component of symbiont-bearing capsules and a critical mediator of symbiont survival during extracellular transmission^42^. While such examples highlight the role of host-derived molecules in symbiont maintenance, their diversity and genetic bases are yet to be explored across most insect lineages despite the prevalence of extracellular transmission routes^40^.

To define the traits involved in sphere production in tortoise beetles, we performed a comparative transcriptomic analysis of foregut symbiotic organs and ovary-associated glands in reproductively active *Chelymorpha alternans*, where egg-laying commences ~29  ±  2.21 days following adult eclosion^33^. Additionally, to provide a robust genomic framework for this analysis, we generated a high-quality reference genome for *C. alternans* using HiFi long-read sequencing (Table S1), the first for a tortoise beetle. Based on genome-guided transcriptome annotation, we identified 1,251 differentially expressed genes (DEGs) between the two tissue-types (false discovery rate [FDR]-adjusted *p* < 0.05), with 502 upregulated in ovary-associated glands (Supplementary Data 1A) and 749 in foregut symbiotic organs (Supplementary Data 1B).

Among the genes with the greatest fold change in the ovary-associated glands was a member of the Yellow family, encoding a protein with a conserved MRJP domain (Fig. 1J). In honey bees, the Yellow protein MRJP1 forms a highly abundant complex with apisimin, which exhibits gelatinous properties and constitutes a major component of royal jelly^9,16^. Given the gel-like features of the spherical secretions that embed *Stammera* (Fig. 1H, I), we posited that Yellow may be similarly abundant within the spheres and contribute to the formation and structural integrity of the surrounding matrix^42,47^.

To test this, we analyzed the protein composition of the symbiont-bearing spheres using sodium dodecyl sulfate polyacrylamide gel electrophoresis (SDS-PAGE). The resulting gel revealed a prominent band closely matching the predicted molecular weight ( ~ 98 kDa) of the candidate Yellow (Fig. 1K). Liquid chromatography-mass spectrometry (LC-MS) confirmed that peptides from this band matched the transcript highly expressed in ovary-associated glands, validating its identity as a Yellow protein (Fig. S1).

### *Yellow* expression is restricted to the ovary-associated glands

**Fig. 2:**
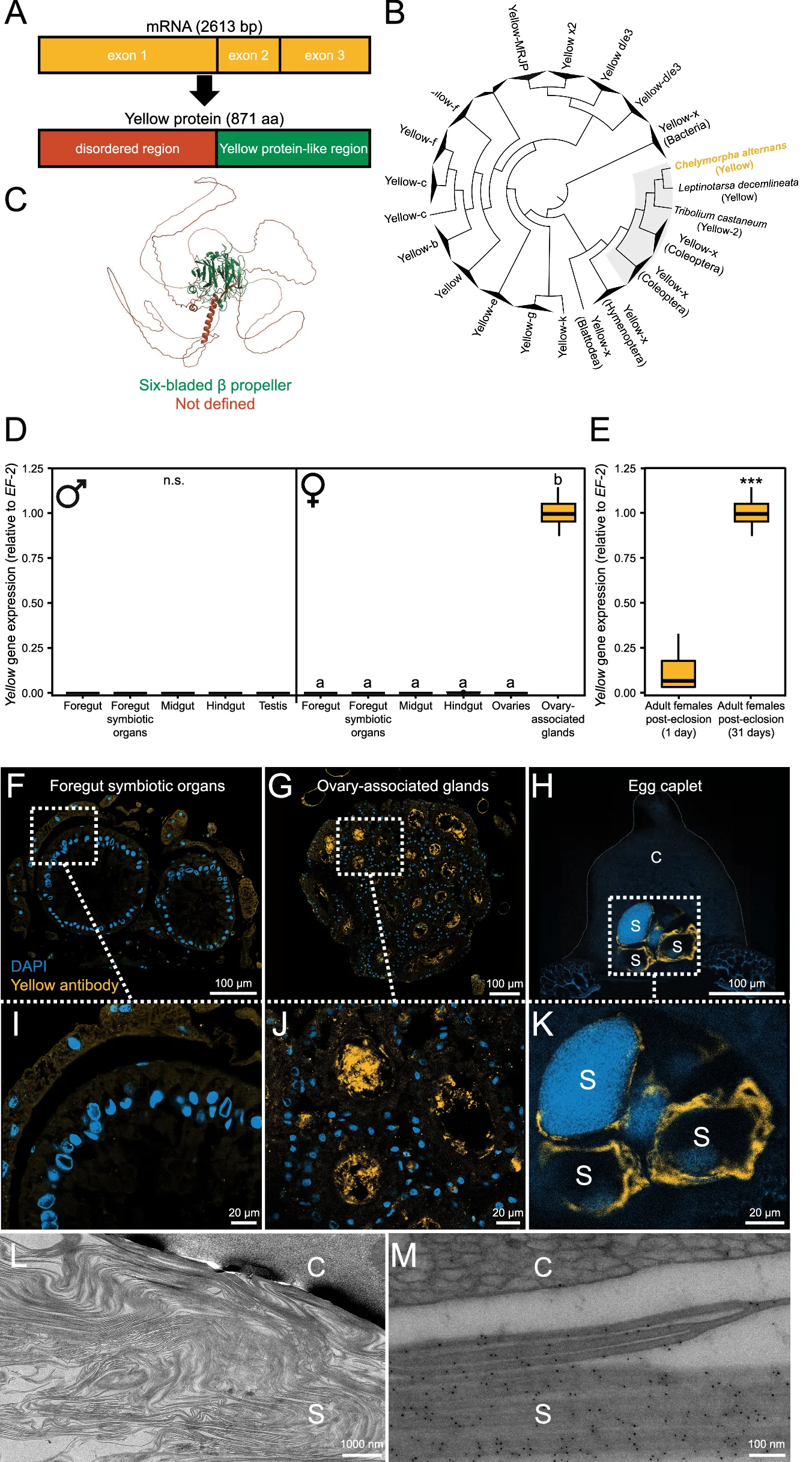
Yellow is produced in the ovary-associated glands and is secreted into the egg caplet, embedding *Stammera.* **A** Gene organization of *yellow*. **B** Maximum Likelihood phylogeny of 269 Yellow protein sequences from diverse insect taxa. A subset of branches is collapsed for simplified visualization. **C** AlphaFold structural prediction of Yellow, with regions color-coded to correspond to gene features shown in (**A**). **D** Gene expression of *yellow* across various male and female body compartments, normalized to the expression in ovary-associated glands of females 31 days following adult eclosion (set to 1). Expression measured by RT-qPCR as the ratio of *yellow* cDNA to *EF-2* cDNA. Different letters above box plots indicate significant differences in female compartments (*n* = 4 biological replicates per stage, two-sided Kruskal-Wallis test, *X*^*2*^ = 19.828, df = 5, *p* = 0.001346). *Yellow* expression was undetectable in all male tissues, confirming its sex-specific expression (two-sided Wilcoxon rank-sum test, *W* = 290, *p* = 0.034). Boxes represent the interquartile range (25th-75th percentiles), lines indicate medians, and whiskers denote range. No significant differences (n.s.). The sex symbols were created in Biorender. (2026) https://BioRender.com/8fd5aa5. **E** *yellow* gene expression in ovary-associated glands at different female developmental stages, relative to females 31 days following adult eclosion (*n* = 4 biological replicates per stage, two-sided *t*-test, df = 6, *p* = 0.00013). Boxes represent the interquartile range (25–75th percentiles), lines indicate medians, and whiskers denote range. **F**–**K** Immunolocalization of Yellow in cross-sections of foregut symbiotic organs (**F**,**I**), ovary-associated glands (**G**,**J**), and egg caplets (**H**,**K**). (yellow: Yellow antibody; DAPI in blue for DNA staining). Caplet outline in (**H**) is manually drawn. **L** Scanning electron microscope image of an egg caplet showing the Yellow protein matrix. **M** Immunogold labelling of an ultrathin-section electron microscopy image of an egg caplet, showing Yellow protein localization via 6 nm gold particles. Scale bars are included for reference. Abbreviations: C, caplet; S, sphere. Micrographs in Fig. 2F–M are from one individual and are shown for illustrative purposes. Further statistical analysis can be found in Supplementary Data 5. Source data are provided as a Source Data file.

The Yellow protein encoded by *C. alternans* is composed of 871 amino acid residues and features a unique N-terminal region encoded by exon 1 (Fig. 2A–C). This region is predicted to be intrinsically disordered and lacks homology to any known protein domains, including those retained by other Yellow proteins in *C. alternans* (Fig. 2A–C). In contrast, exons 2 and 3 encode a conserved six-bladed β propeller structure characteristic of the MRJP/Yellow-related protein family^8^, as supported by sequence homology and AlphaFold structural predictions (Fig. 2A, C).

Phylogenetic analysis placed the *C. alternans* Yellow protein within the Yellow-x clade (Yellow-x-a) (Fig. 2B and S2), alongside two additional paralogs from the same species (Yellow-x-b and Yellow-x-c) (Fig. S2). Yellow-x is the closest insect clade to bacterial Yellow homologs and is hypothesized to have originated via horizontal gene transfer (HGT) from bacteria^3^. This gene lineage likely underwent multiple rounds of duplication and rapid sequence divergence prior the diversification of insects^3^. Although Yellow-x remains functionally uncharacterized, its closest known homolog in the beetle *Tribolium castaneum* (*TcY-2*) is expressed during pupal and adult stages, with highest levels detected in the elytron (i.e. the modified and hardened forewing)^48^.

We investigated the expression dynamics of *yellow* across different tissue types and sexes in reproductively active *C. alternans* using reverse transcription-quantitative PCR (RT-qPCR). Expression profiling revealed that *yellow* is specifically expressed in the ovary-associated glands of adult females, with negligible expression detected in other anatomical compartments, including the foregut, foregut symbiotic organs, midgut, hindgut, and ovaries (Fig. 2D, right panel; Kruskal-Wallis test, *X*^*2*^ = 19.828, d.f. = 5, *p* = 0.001346). No expression was detected in any male tissues, underlining the sex-specific nature of *yellow* expression (Fig. 2D, left panel; two-sided Wilcoxon rank-sum test, *W* = 290, *p* = 0.034).

Temporal profiling further showed that *yellow* is expressed as early as 1 day following adult eclosion in females, with transcript levels increasing substantially by day 31 (Fig. 2E; two-sided *t*-test, d.f. = 6, *p* < 0.001). This expression trajectory coincides with the onset of reproductive maturation and ovipositing, which begin 24 and 29 days after adult eclosion, respectively^33^. Such findings revealed a specialized role for *yellow* in tortoise beetles, distinct from its well-established function in melanin production along insect wings and cuticles, and potentially analogous to the secretory function of major royal jelly proteins in honey bees^12,49^.

To validate that Yellow is preferentially secreted within the ovary-associated glands, we next examined the protein’s localization by immunostaining on paraffin-embedded cross-sections. By raising an antibody specifically targeting the exon 1-encoded region (Figure S3), we found that Yellow is extracellularly secreted within the ovary-associated glands (Figs. 2G and 2J), where its substantial accumulation contrasts the low signal in the foregut symbiotic organs (Fig. 2F, I). These findings are highly consistent with tissue-specific expression dynamics for the Yellow-encoding gene. Additionally, Yellow was observed within the egg caplets, encasing *Stammera* (Fig. 2H, F). To validate these observations at a higher resolution, we performed immunogold labelling on ultrathin-sections followed by transmission electron microscopy. This approach further confirmed the localization of Yellow and revealed that the protein forms layers of sheets, defining the spheres (Fig. 2L, M), consistent with its embedding role for *Stammera* during extracellular transmission^28,30,33^.

### Molecular evolution of Yellow across tortoise beetles

**Fig. 3:**
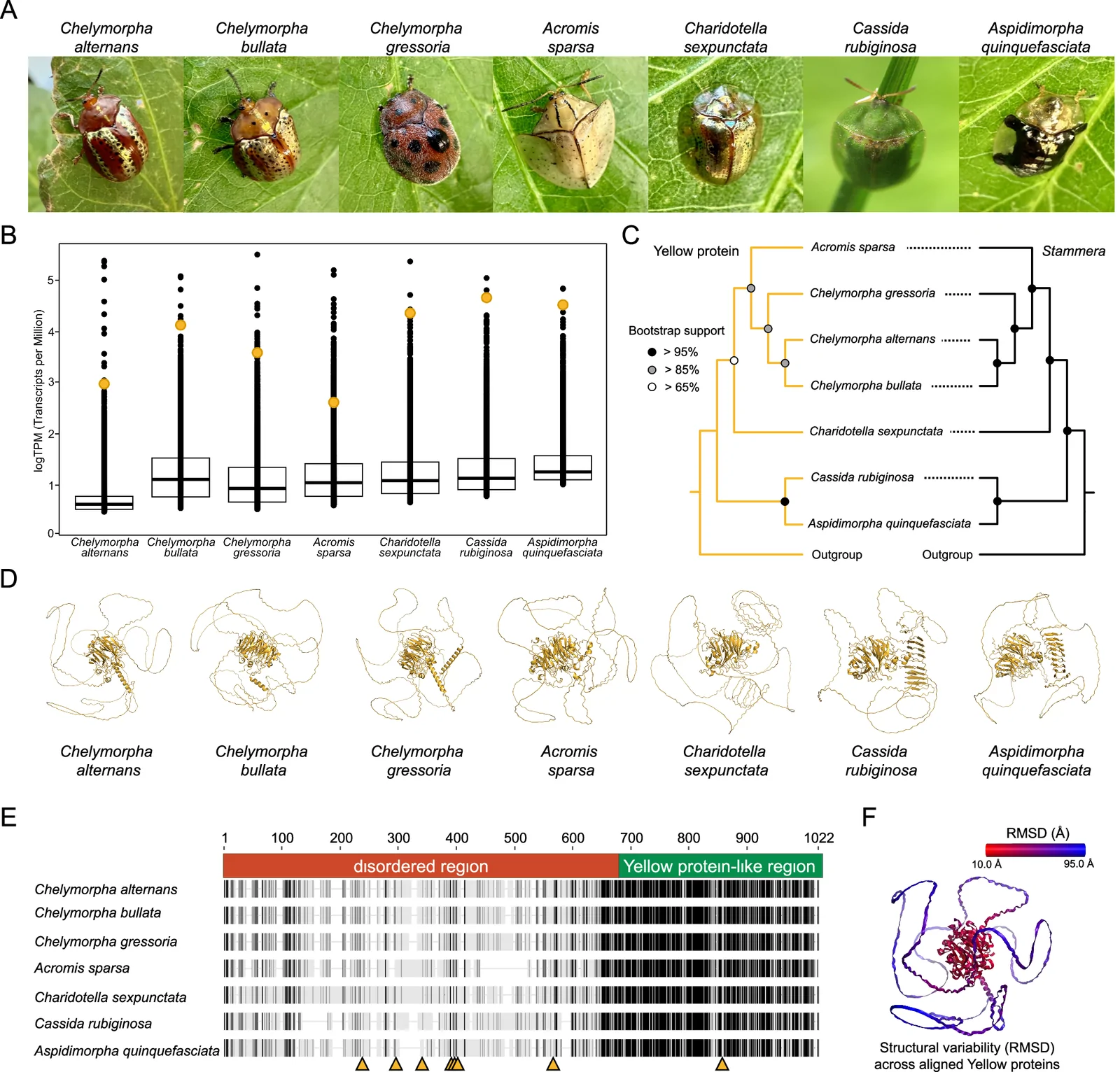
Molecular evolution of Yellow in tortoise beetles. **A** Seven tortoise beetle species. **B** Transcript abundance (logTPM) from ovary-associated gland transcriptomes of the depicted species. Boxes represent the interquartile range (25–75th percentiles), and horizontal lines indicate medians. Whiskers extend to the most extreme values within 1.5 x the interquartile range. Individual points represent transcript-level abundance values, and yellow circles indicate Yellow-encoding transcripts. For each species, one biological sample (a single pair of ovary-associated glands) was sequenced. Variation shown reflects transcript-level distribution within a species. **C** Tanglegram illustrating co-cladogenesis between Yellow proteins and *Stammera* lineages. Maximum Likelihood phylogenies were inferred from an alignment of Yellow protein sequences and a concatenated alignment of 61 core proteins for the symbiont phylogeny. **D** AlphaFold structural predictions of Yellow proteins from Cassidinae species. **E** Alignment of Yellow protein sequences from seven Cassidinae species representing three tribes, showing positively selected sites that are enriched in the disordered region encoded by exon 1 (one-sided Fisher’s Exact Test, *p* < 0.05). **F** Root Mean Square Deviation (RMSD)-based structural variability mapped onto the *C. alternans* Yellow protein structure. RMSD values were calculated by aligning six Yellow protein structures to *C. alternans* and measuring per-residue Cα atom deviations. Values were mapped onto the reference structure using a red-blue color gradient, where red indicates low structural variability and blue indicates high variability. Further statistical analysis can be found in Supplementary Data 5.

In tortoise beetles, egg-associated spheres represent a shared and conserved symbiont transmission strategy^28,29,34,50^. To validate whether Yellow is similarly featured in sphere production across other members of the Cassidinae subfamily, we analyzed the transcriptomes of ovary-associated glands from six additional tortoise beetle species spanning three tribes: *Chelymorpha bullata* (tribe: Mesomphaliini), *Chelymorpha gressoria* (tribe: Mesomphaliini), *Acromis sparsa* (tribe: Mesomphaliini), *Charidotella sexpunctata* (tribe: Cassidini), *Cassida rubiginosa* (tribe: Cassidini), and *Aspidimorpha quinquefasciata* (tribe: Aspidimorphini) (Fig. 3A and Table S2). Similar to *C. alternans* (Figs. 1, 2), a y*ellow* homolog ranked among the most highly expressed genes compared to the median transcript expression within the transmission organs across all species (Fig. 3B), and the corresponding proteins were subsequently validated in symbiont-bearing spheres via SDS-PAGE and LC-MS analysis (Fig. S4 and Table S3).

Comparative protein sequence and structural analyses revealed a conserved β-propeller domain and a consistently variable, intrinsically disordered N-terminal region, reflecting an organization analogous to that observed in *C. alternans* (Fig. 3D and S5). To quantify structural variability across species, we performed a per-residue root-mean-square deviation (RMSD) analysis using superimposed AlphaFold models^51,52^. RMSD values were mapped onto the *C. alternans* structure, revealing that the disordered N-terminal region displays elevated conformational flexibility, while the C-terminal β-propeller remains structurally conserved across species (Fig. 3F). Notably, regions with high RMSD values often coincided with low model confidence scores (pLDDT), indicating that both sequence divergence and reduced structural predictability contribute to apparent variability in the N-terminal domain (Figs. S5, 6 and Table S4).

Codon substitution analysis using Fixed Effects Likelihood (FEL)^53^ indicated that the N-terminal region is enriched for positively selected sites (Fisher’s Exact Test, *p* < 0.05) (Fig. 3E), while the C-terminal β-propeller domain is under strong purifying selection (Fisher’s Exact Test, *p* < 0.001), encompassing 165 conserved residues (Fig. S7 and Supplementary Data 2). Structural modeling further indicated that the β-propeller may harbor buried hydrophobic residues, consistent with a stabilizing core and shared with other Yellow and MRJP proteins^8^. In contrast, the disordered N-terminal region features exposed potential hydrophobic patches, with positively selected residues clustering in these areas, likely contributing to functional divergence (Fig. S8). These results support a dual-function model in which the conserved C-terminal domain acts as a structural scaffold, while the rapidly evolving N-terminal region acts as a flexible interface potentially involved in matrix formation or symbiont surface recognition. This organization may reflect strong functional constraints on the core domain, paired with a potential hotspot for evolutionary innovation concentrated in the disordered N-terminus.

Consistent with this model, the phylogeny of the identified Yellow proteins closely mirror that of *Stammera* (Fig. 3C), supporting a co-diversified evolutionary history between tortoise beetles and their symbionts^29,34^. In line with this, Pons and colleagues^36^ recently demonstrated that experimental cross-infection of symbionts across tortoise beetles resulted in failed vertical transmission. Non-native *Stammera* strains were not inherited by offspring, and only sterile spheres, composed of host-derived Yellow, were secreted^36^. This outcome highlights the specificity and molecular basis required for successful vertical transmission, suggesting that each Yellow protein may have evolved to interact specifically with its corresponding *Stammera* species, coating the symbiont and embedding it within the egg caplet.

### *Yellow* expression along the ovary-associated glands

Female accessory glands are widespread secretory structures associated with the insect reproductive tract^54^. While traditionally linked to oviposition, producing egg coatings, adhesives, and lubricants, they also display remarkable functional features and developmental origins across taxa^54,55^. Their secretions may include proteins, carbohydrates, lipids, pheromones, antimicrobial agents, and nutrient-rich substances^54,56^. In several insect groups, accessory glands have also been shown to underpin vertical transmission of microbial symbionts by producing extracellular matrices or milk-like substances that encapsulate, coat, or nourish symbionts during transfer to the next generation^40,42,57–62^. For example, urostylidid bugs embed and extracellularly transmit their obligate nutritional symbiont, *Tachikawaea gelatinosa*, by secreting a galactan-rich jelly^41^. That matrix is consumed by newly hatched larvae, which initiates infection by *Tachikawaea*^41^. Specialized anatomical traits account for jelly production in urostylidid bugs, including female-specific organs associated with the genital chamber^41^. In tortoise beetles, the ovary-associated glands represent similarly specialized organs for symbiont transmission. But within these organs, distinct anatomical regions may coordinate distinct tasks, including symbiont maintenance and proliferation, relative to Yellow expression and secretion.

**Fig. 4:**
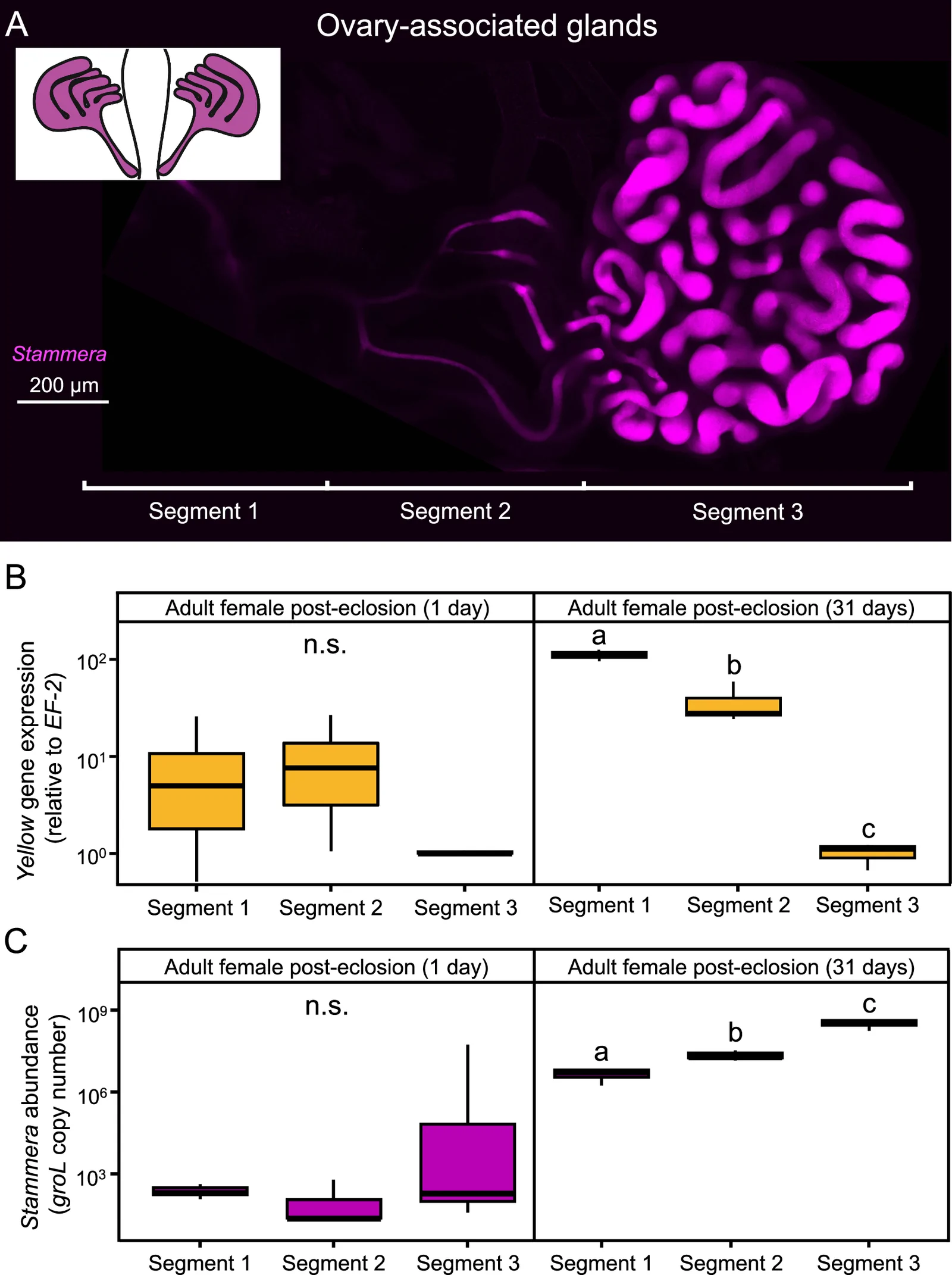
*Stammera* proliferation in the ovary-associated glands is decoupled from Yellow production. **A** Whole-mount of an ovary-associated gland showing the distinct segments analyzed for *yellow* gene expression. *Stammera* is visualized via FISH (magenta: 16S rRNA). The figure illustrating the ovary-associated glands was created in Biorender. García-Lozano, M. (2026) https://BioRender.com/sg2u5l8. **B** Left: *yellow* expression across different ovary-associated gland segments in females 1 day following adult eclosion. Expression levels were quantified by RT-qPCR and are presented as the ratio of *yellow* cDNA to *EF-2* cDNA. The average expression in Segment 3 is set to 1. No significant differences were detected among segments (n.s.) (*n* = 3 biological replicates per segment; linear model (LM), ANOVA: *F*_(2,4)_ = 2.9, *p* = 0.1626). Right: *yellow* expression across the same segments in females 31 days following adult eclosion. Expression values are normalized to Segment 3 (set to 1). Significant differences across segments were detected (*n* = 3 biological replicates per segment; linear model (LM), ANOVA: *F*_(2,4)_ = 69.5, *p* = 0.0007816), and post hoc pairwise comparisons were performed using Tukey contrasts with Bonferroni adjustment for multiple comparisons. Different letters above box plots indicate statistically significant differences. Boxes represent the interquartile range (25th-75th percentiles), lines indicate medians, and whiskers denote range. **C** Left: Symbiont abundance across ovary-associated gland segments in females 1 day following adult eclosion. Symbiont titers are expressed as *groL* copy numbers from qPCR. No significant differences were detected among segments (n.s.) (*n* = 3 biological replicates per segment; linear model (LM), ANOVA: *F*_(2,4)_ = 0.7, *p* = 0.5265). Right: Symbiont abundance across ovary-associated gland segments in females 31 days following adult eclosion. Significant differences across segments were detected (*n* = 3 biological replicates per segment; linear model, ANOVA: *F*_(2,4)_ = 49.2, *p* = 0.001523) followed by post hoc pairwise comparisons using Tukey contrasts with Bonferroni adjustment for multiple comparisons). Different letters above box plots indicate statistically significant differences. Boxes represent the interquartile range (25–75th percentiles), lines indicate medians, and whiskers denote range. Further statistical analysis can be found in Supplementary Data 5. Source data are provided as a Source Data file.

To uncover the stepwise process by which *Stammera* becomes encased in Yellow, we analyzed the spatial expression of *yellow* relative to symbiont abundance in the ovary-associated glands of *C. alternans*, subdividing these structures into three anatomically distinct segments (Fig. 4A).

In reproductively immature females (1 day after adult eclosion), *yellow* expression did not differ significantly among segments (Fig. 4B, left panel; LM, *F*_(2,4)_ = 2.9, *p* = 0.16), indicating an absence of spatial patterning at this early reproductive stage. By contrast, reproductively active, egg-laying females (31 days after adult eclosion) exhibited elevated *yellow* expression in Segment 1, followed by Segment 2, while Segment 3 consistently showed low expression (Fig. 4B, right panel; LM, *F*_(2,4)_ = 69.5, *p* = 0.0007816). The observed expression pattern suggests that *yellow* may be preferentially restricted to the gland’s proximal regions as females mature.

*Stammera* abundance was uniformly low across all segments of the ovary-associated glands in reproductively immature females (Fig. 4C, left panel; LM, *F*_(2,4)_ = 0.7, *p* = 0.52). In contrast, mature females showed markedly higher *Stammera* abundance in Segment 3 (Fig. 4C, right panel; LM, *F*_(2,4)_ = 49.2, *p* = 0.001523), despite consistently low *yellow* expression in this region. The uncoupling of *yellow* expression from symbiont abundance in Segment 3 suggests that symbiont maintenance and transmission are governed by distinct molecular programs within the symbiotic gland. This spatial partitioning likely reflects functional specialization: distal regions (i.e. Segment 3) may serve as sites of symbiont proliferation and storage, whereas proximal segments (i.e. Segments 1 and 2) are dedicated to *yellow* expression and matrix production for transmission. Under this model, *Stammera* may proliferate along the distal ends of the ovary-associated glands, subsequently becoming encapsulated by Yellow as it is secreted beyond these structures.

### A novel symbiotic organ for sphere storage

**Fig. 5:**
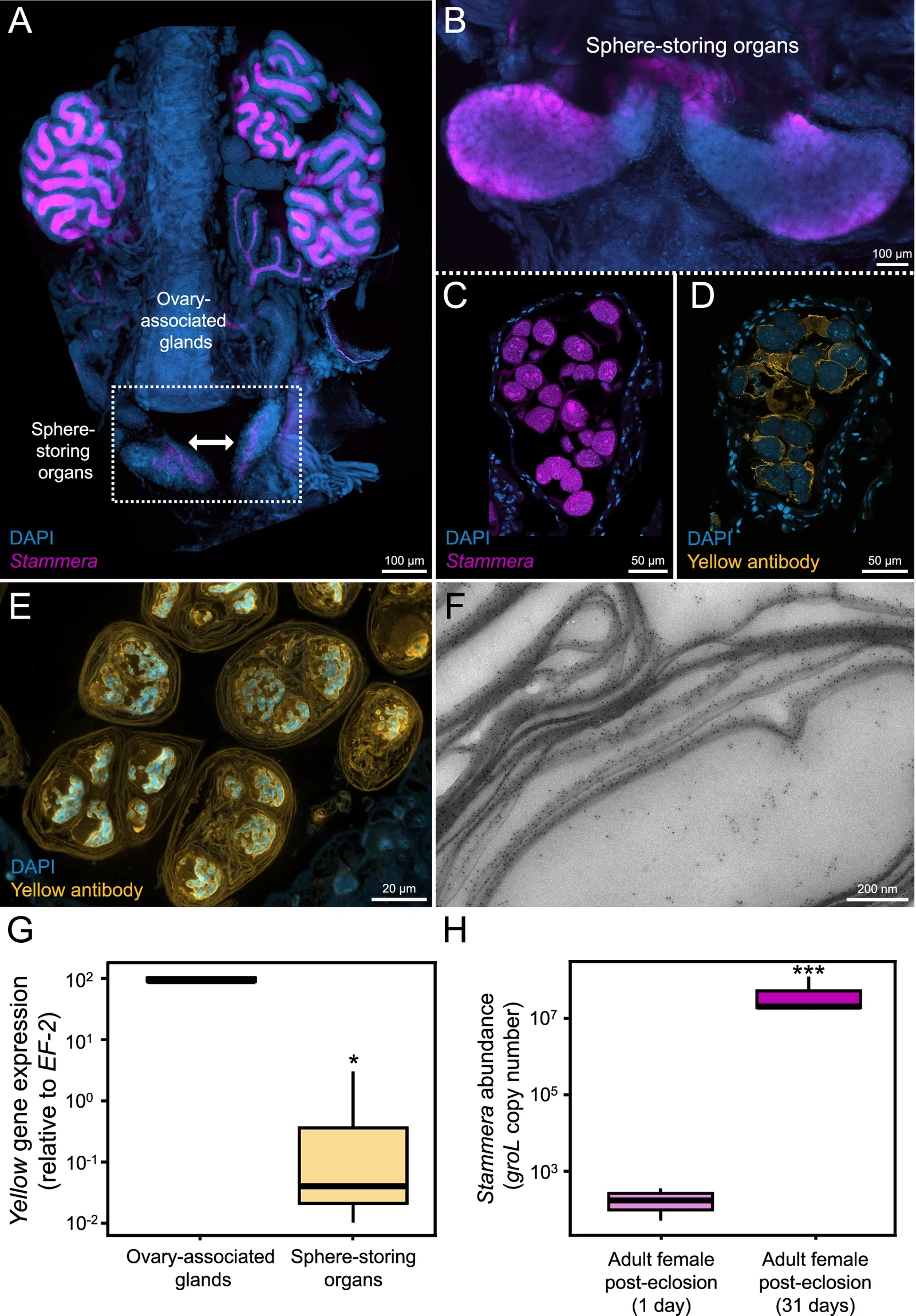
Spheres are stored in a novel symbiotic organ prior to release. **A**,**B** Whole-mount fluorescence in situ hybridization (FISH) of ovary-associated glands and the newly identified symbiont-storing organ in an adult female, targeting *Stammera* (magenta: 16S rRNA). DAPI (blue) was used for DNA staining. **C**,**D** Cross-sections of the symbiont-storing organ showing FISH (**C**) targeting *Stammera* (magenta: 16S rRNA) and immunostaining (**D**) for the Yellow protein (yellow: Yellow antibody). **E** Thin-section immunostaining of the symbiont-storing organ showing localization of Yellow (yellow). DAPI (blue) used for DNA staining. **F** Immunogold-labelled ultrathin section of the symbiont-storing organ visualized by electron microscopy. Yellow protein localization is visualized via a secondary antibody conjugated to 6 nm gold particles. Scale bars are provided for reference. **G** *Yellow* gene expression in symbiont-storing organs compared to ovary-associated glands in females 31 days following adult eclosion. RT-qPCR data show *yellow* cDNA levels normalized to *EF-2* cDNA; values are expressed relative to ovary-associated glands. Asterisks indicate significance (*n* = 3 biological replicates per tissue type, two-sided *t*-test, df = 4, *p* = 0.0169). Boxes represent the interquartile range (25th-75th percentiles), lines indicate medians, and whiskers denote range. **H** *Stammera* abundance in symbiont-storing organs of females 1 day versus 31 days following adult eclosion, quantified via copy number of the *groL* gene. Asterisks indicate significance (*n* = 3 biological replicates per tissue type, two-sided *t*-test, df = 4, *p* = 0.000149). Boxes represent the interquartile range (25–75th percentiles), lines indicate medians, and whiskers denote range. Micrographs in Fig. 5A–F are from one individual and are shown for illustrative purposes. Further statistical analysis can be found in Supplementary Data 5. Source data are provided as a Source Data file.

Sphere allocation is highly precise in tortoise beetles: females deposit an average of ~12 ( ± 2.3) spheres per egg^30^. How this level of calibration is achieved mechanistically remains unresolved, particularly regarding the destination of the *Stammera*-bearing spheres before release. Towards addressing this, we identified a third, and previously undescribed, symbiotic organ; one that is exclusively dedicated to sphere storage (Fig. 5).

Here, we discovered a pair of bulbous sacs located beneath, and connected to, the ovary-associated glands (Fig. 5A). Light and electron microscopy revealed that they solely contain the symbiont-bearing spheres. Whole-mount fluorescence in situ hybridization (FISH) confirmed a strong *Stammera* signal within these globular sacs (Fig. 5B). FISH on tissue cross-sections showed that the spheres are enclosed by a single, thin epithelial layer of host cells (Fig. 5C). Immunostaining of adjacent sections demonstrated that the spheres are coated with Yellow, as previously observed in egg caplets (Fig. 5D). Immunogold labeling further revealed that Yellow forms a dense matrix around the spheres, consistent with its ultrastructural localization in the egg caplets (Fig. 5E, F). This indicates that the structure functions as a temporary reservoir for symbiont-bearing spheres prior to deposition.

To determine whether Yellow is synthesized within the sphere-storing organ, we performed RT-qPCR on dissected tissues. *Yellow* transcript levels were nearly undetectable, in contrast to high expression in ovary-associated glands (Fig. 5G; two-sided *t*test, d.f. = 4, *p* = 0.0169), indicating that the spheres are already formed prior to storage within these organs. Symbiont titer measurements further demonstrated that this third organ is filled with *Stammera*-bearing spheres in reproductively active females but remains empty in newly emerged, reproductively immature beetles (Fig. 5H; two-sided *t*-test, d.f. = 4, *p* = 0.000149).

The spatial separation of Yellow synthesis and sphere accumulation indicates a coordinated, multi-organ strategy for symbiont packaging and transmission. Unlike the ovary-associated glands, which produce and secrete the protein matrix and the symbiont cells (Fig. 4), the sphere-storing organ appears to function exclusively as a holding chamber (Fig. 5).

This anatomical division contrasts with other symbiotic systems in insects. For example, in plataspid stinkbugs, both symbionts and matrix components are housed in a single compartment, and symbiont capsules are directly secreted through the hindgut during oviposition^42^. In tortoise beetles, by contrast, spheres are produced in one organ, accumulate in another, ahead of being allocated to each individual egg^30^. This spatial and functional separation suggests that the sphere-storing organ serves as a staging site, allowing the host to potentially regulate the timing of sphere deposition as it coincides with egg laying. Having characterized the stepwise process of Yellow synthesis and sphere production, its embedding of *Stammera* and storage ahead of transfer, we next asked: what is the protein’s functional role during symbiont transmission?

### Functional knockdown of Yellow confirms its structural role in symbiont encapsulation

**Fig. 6:**
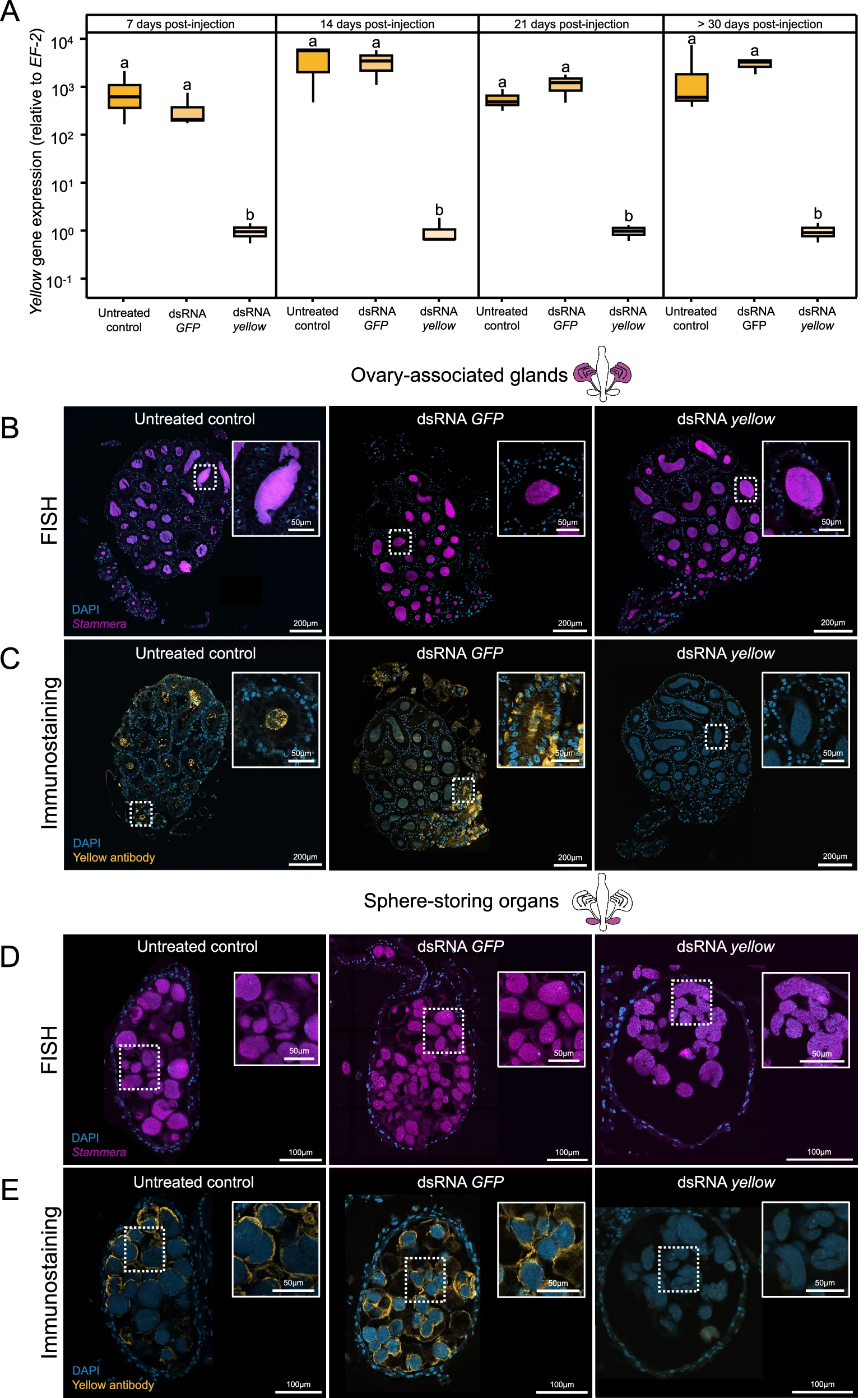
RNA interference effectively reduces *yellow* expression and depletes its encoded protein in tortoise beetles. **A** *Yellow* expression levels from week 1 to week 5 post-injection in untreated controls, dsRNA-*GFP*, and dsRNA-*yellow* groups. Expression was quantified by RT-qPCR and is shown as the ratio of *yellow* cDNA to *EF-2* cDNA. Expression levels of *yellow* were significantly reduced in dsRNA *yellow* injected females at timepoint 1 (*n* = 3 biological replicates per treatment; linear model (LM), ANOVA: *F*_(2,4)_ = 65.08, *p* = 0.0008), timepoint 2 (*n* = 3 biological replicates per treatment, LM, ANOVA: *F*_(2,4)_ = 78.005, *p* = 0.0006), timepoint 3 (*n* = 3 biological replicates per treatment, LM, ANOVA: *F*_(2,4)_ = 126.11, *p* = 0.0002), and timepoint 4 (*n* = 3 biological replicates per treatment, LM, ANOVA: *F*_(2,4)_ = 94.09, *p* = 0.0004) compared to untreated and dsRNA *GFP*-injected females. Post hoc pairwise comparisons were performed using Tukey contrasts with Bonferroni adjustment for multiple comparisons. Different letters above box plots indicate significant differences. Boxes represent the interquartile range (25th-75th percentiles), lines indicate medians, and whiskers denote range. **B** Cross-section fluorescence in situ hybridization (FISH) of ovary-associated glands from females in different treatment groups, 5 weeks post-injection, targeting *Stammera* (magenta: 16S rRNA). **C** Immunostaining of adjacent cross-sections from the same samples, targeting Yellow (yellow: Yellow antibody). The figure illustrating the ovary-associated glands was created in Biorender. García-Lozano, M. (2026) https://BioRender.com/kjqn4kh. **D**,**E** Cross-sections of the symbiont-storing organ from untreated controls, dsRNA-*GFP*, and dsRNA-*yellow* treatments showing FISH (**D**) for *Stammera* (magenta: 16S rRNA) and immunostaining (**E**) for Yellow (yellow: Yellow antibody). The figure illustrating the sphere-storing organs was created in Biorender. García-Lozano, M. (2026) https://BioRender.com/n3lg8w2. DAPI (blue) was used in all panels for DNA staining. Scale bars are included for reference. RNA interference experiments were repeated independently at least 3 times with similar results. Micrographs in 6B-6E are shown for illustrative purposes. Further statistical analysis can be found in Supplementary Data 5. Source data are provided as a Source Data file.

To test the functional importance of Yellow during symbiont transmission, we employed RNA interference (RNAi) in the tortoise beetle *C. alternans*. We synthetized double-stranded RNA (dsRNA) targeting exon 1 of *yellow*, given its uniqueness, and injected it into adult females. Control groups included individuals injected with dsRNA against green fluorescent protein (GFP) and non-injected females. RT-qPCR analysis of ovary-associated glands from week 1 to week 5 post-injection confirmed a > 1,000-fold reduction in *yellow* transcript relative to controls as early as week 1 (Fig. 6A; LM, *F*_(2,4)_ = 65.08, *p* = 0.0008). This suppression persisted throughout the 5-week time course (Fig. 6A; LM, *F*_(2,4)_ = 94.09, *p* = 0.0004), demonstrating that RNAi is both efficient and stable in tortoise beetles, consistent with previous findings in other coleopteran species^63–67^.

**Fig. 7:**
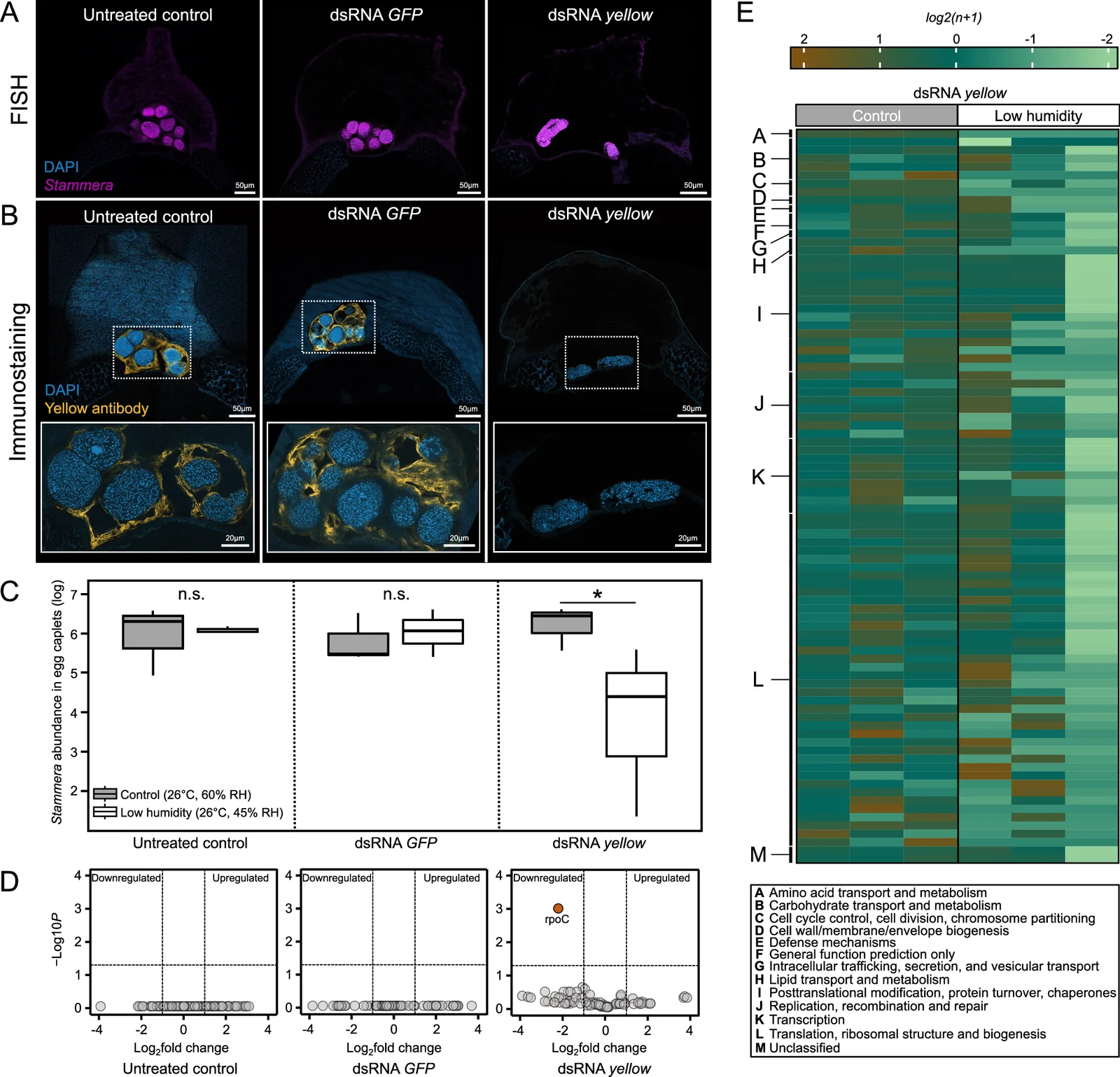
Yellow protects *Stammera* from desiccation during extracellular transmission. **A** Cross-section fluorescence in situ hybridization of eggs laid by females from different treatment groups, targeting *Stammera* (magenta: 16S rRNA). DAPI (blue) was used for DNA staining. Micrographs are from one single individual and are shown for illustrative purposes. **B** Sections from individuals form the same experiment showing Yellow protein localization (yellow: Yellow antibody). The egg caplet outline in the dsRNA-*yellow* panel is manually drawn. Scale bars are included for reference. RNA interference experiments were repeated independently at least three times with similar results. Replicates for the dsRNA-yellow treatment are shown in Fig. S10; the untreated control and dsRNA-*GFP* treatments shown here serve as experimental controls. **C** *Stammera* titers in eggs from females during the first two weeks post-injection under both control and low humidity conditions, quantified as *groL* gene copy number (*n* = 3 biological replicates per treatment). Differences between control and low humidity treatments were assessed using negative binomial generalized linear models (NB-GLM; two-sided Wald tests). Different letters above box plots indicate significant differences. There was no difference in symbiont titers of eggs from untreated females (NB-GLM, *p* = 0.5) and dsRNA *GFP*-injected females (NB-GLM, *p* = 0.67) under low humidity conditions. Eggs from dsRNA *yellow*-injected females showed lower symbiont titers under low humidity (NB-GLM, *p* = 0.0315). Boxes represent the interquartile range (25–75th percentiles), lines indicate medians, and whiskers denote range. **D** Volcano plots comparing gene expression under control vs. low humidity conditions across untreated, dsRNA *GFP*, and dsRNA *yellow* treatments (*n* = 3 biological replicates per treatment). Differential expression was analyzed separately for each treatment group using DESeq2 (negative binomial generalized linear model). Significance was assessed using two-sided Wald tests, with Benjamini-Hochberg false discovery rate (FDR) correction for multiple comparisons across genes. **E** Heatmap of *Stammera* gene expression in eggs from dsRNA-*yellow* females under control and low humidity conditions. Further statistical analysis can be found in Supplementary Data 5. Source data are provided as a Source Data file.

To evaluate the effects of *yellow* knockdown at the tissue level, we performed FISH and immunostaining on cross-sections of ovary-associated glands (Fig. 6B, C) and sphere-storing organs (Fig. 6D, E). FISH revealed that *Stammera* remained detectable across all treatment groups, indicating that Yellow is not required for symbiont localization or persistence within host tissues (Fig. 6B). Immunostaining, however, confirmed the pronounced and conspicuous depletion of Yellow in the knockdown individuals (Fig. 6C), consistent with the observed transcript reduction (Fig. 6A). Notably, symbiont-bearing structures within the sphere-storing organ of *yellow*-knockdown females exhibited irregular, elongated morphology (Fig. 6D, E). Similarly, eggs laid by knockdown individuals contained fewer of these secretions, which also appeared malformed (Fig. 7A). Immunostaining confirmed the absence of Yellow coating in eggs caplets, underscoring its essential role in maintaining the structure and integrity of the symbiont-embedding spheres (Figs. 6E, 7B).

The morphological defects observed in *yellow*-knockdown spheres demonstrated that Yellow is essential for matrix integrity (Figs. 6E, 7B). This phenotype aligns with structural predictions: surface-exposed hydrophobic patches in its disordered N-terminal region and a conserved hydrophobic cavity within the β-propeller domain (Fig. S8). Glycosylation detected by mass spectrometry likely enhances protein stability or solubility in the extracellular environment and may interact with hydrophobic domains to promote matrix formation (Fig. S1B)^68,69^. These molecular features support a model in which both hydrophobic and glycosylation-mediated interactions are required to assemble or stabilize the protective matrix.

This is reminiscent of MRJP-rich royal jelly in honey bees, where glycosylated MRJP1 forms in complex with apisimin, a gelatinous matrix that supports larval adhesion and positioning within vertically oriented queen cells^16^.

Other proteins have been similarly adapted for structural roles in symbiont encapsulation across insects^42,43,47^. In plataspid stinkbugs, an odorant-binding protein is secreted by female accessory glands to form a capsule-like structure that encloses gut symbionts during vertical transmission^42^. Likewise, in the stinkbug *Plautia stali*, a mucin-like glycoprotein produced in the female-specific symbiotic organ is smeared along with the symbiotic bacteria, suggesting a role mitigating the abiotic stresses that accompany extracellular transfer^47^. Together, these parallels point to convergent strategies, in which diverse proteins have been independently recruited as protective scaffolds for extracellular symbiont transfer^40–42,47^. Since *Stammera* can persist in the absence of Yellow while in the ovary-associated glands of its host, we next asked whether its embedding properties and structural encapsulation protect the microbe from environmental stressors during extracellular transmission.

### Yellow is required for symbiont stability under abiotic stress

We previously reported that *Stammera* preferentially expresses stress-tolerance genes while residing in the egg caplet^34^, indicating that it modulates its metabolism to endure the metabolic challenges of extracellular transmission. This aligns with the abiotic challenges encountered during transfer^40,42^, including desiccation, osmotic shock, and temperature fluctuations^70–73^.

In tortoise beetles, *Stammera* must persist for up to 11 days outside host tissues before being acquired in the final day of embryogenesis^30,33^. Given Yellow’s role in forming a protective matrix (Fig. 2), and its gelling properties in other insect groups (e.g. honeybees)^16^, we hypothesized that it may enhance *Stammera*’s ability to withstand desiccation during extracellular transfer. To test this, we exposed eggs from Yellow-knockdown and control females to two relative humidity conditions (control: 60%; low: 45%) and quantified *Stammera* titers. These humidity levels encompass environmental conditions within the native range of the *C. alternans* in the Republic of Panama, Costa Rica, Colombia and Brazil^74,75^.

By day 9 post-oviposition, symbiont titers were significantly reduced in eggs from *yellow* knockdown females reared under low humidity conditions (Fig. 7C; NB-GLM, *z* = 2.15, *p* = 0.0315), while both control groups maintained stable *Stammera* levels (Fig. 7C; untreated control, NB-GLM, *z* = 0.66, *p* = 0.506; dsRNA *GFP*, NB-GLM, *z* = −0.41, *p* = 0.679). The observed reduction demonstrates that Yellow promotes symbiont survival during environmental stress, likely by buffering the spheres against water loss and associated cellular damage. This hydrating role mirrors findings in other insects: in the red flour beetle *Tribolium castaneum*, Yellow contributes to adult cuticle waterproofing^76^, while in the Asian tiger mosquito *Aedes albopictus*, Yellow proteins are essential for egg desiccation resistance^19,20^. Such examples reinforce Yellow’s broader role as an extracellular protectant across diverse physiological contexts and highlight its co-option by tortoise beetles to sustain their symbionts during extracellular transfer.

Finally, we asked whether *Stammera* mounts a compensatory response to stress in the absence of Yellow by performing RNA-seq on egg samples, allowing us to compare symbiont transcriptomes across treatments under control (60% RH) and low humidity conditions (45% RH). No differentially expressed genes were detected between treatments in the control groups (Fig. 7D and Supplementary Data 3). Under low humidity, however, we observed the striking and significant downregulation of *rpoC* in Yellow-deficient eggs (Fig. 7D). *RpoC* encodes the β′ subunit of RNA polymerase, a core component of the transcriptional machinery (Fig. 7D)^77^. In *Escherichia coli*, mutations in *rpoC* reduce transcriptional activity and impair adaptation to osmotic stress^77^. Consistent with this, global transcriptomic activity was broadly reduced in Yellow-deficient eggs under low humidity (Fig. 7E). Correspondingly, a larger proportion of genes showed fold-change values below 1, indicating widespread downregulation in Yellow-deficient eggs (Fig. S9, Fisher’s Exact Test, *p* < 0.01).

Under dehydrating conditions, water loss elevates intracellular solute concentration, creating osmotic imbalance that destabilizes proteins and disrupts enzyme function^78,79^. Such mechanistic insights align with our findings, revealing that in the absence of Yellow, unbuffered desiccation stress triggers transcriptional collapse in *Stammera*, with *rpoC* downregulation marking the failure of its core gene expression machinery (Fig. 7D).

Our results indicate that Yellow is essential not only for the structural encapsulation of *Stammera* within spheres consumed by developing embryos, but also for preserving its transcriptional and metabolic activity under environmental stress. Despite prior evidence that *Stammera* expresses stress-tolerance genes during transmission^34^, our data indicate its intrinsic defenses are insufficient without host-derived protection. Here, Yellow underpins the gelling matrix critical for sustaining an obligate symbiont during extracellular transmission.

Our study details how an ancient insect gene family has been co-opted to stabilize extracellular symbionts, broadening the functional characterization of Yellow proteins and uncovering additional mechanistic insights into how insects compensate for the reduced metabolism of their symbionts during vertical transfer. In tortoise beetles, a female-specific *yellow* gene encodes a glycosylated, matrix-forming protein that forms the symbiont-bearing spheres during vertical transmission. Disrupting *yellow* (i) impairs matrix integrity, (ii) disrupts sphere morphology, (iii) reduces symbiont viability under desiccation, (iv) and triggers transcriptional collapse in *Stammera*, marked by *rpoC* downregulation, a key determinant of tolerance to osmotic stress^77^. Our findings establish Yellow as a critical extracellular scaffold in tortoise beetles, paralleling the structural role of MRJP1 in honeybees^3,12,15,16^.

Phylogenetic congruence between *yellow* and *Stammera* supports long-term co-diversification between symbiont and host^28,29,34,36,37^, suggesting reciprocal adaptation to ensure transmission fidelity, inviting future studies on cross-species matrix compatibility and the molecular interface between symbiont and host. More broadly, our findings emphasize that protein matrices are a recurring solution to the challenges of extracellular symbiosis, complementing recent findings in stinkbugs and other insects^40,42,43,47^.

Our findings highlight the extraordinary functional diversity of Yellow proteins in insects, extending their known roles in pigmentation^2,5,6,13^, behavior^12,16^, and immunity^8^ to a previously unrecognized function in symbiont stabilization. By forming a glycosylated matrix that embeds *Stammera*, Yellow ensures vertical transmission across generations despite the bacterium’s reduced genome and extracellular lifestyle^28–34,36^.

## Methods

### Insect rearing

*Chelymorpha alternans* beetles used in this study for genome sequencing, organ-specific RNA sequencing (RNA-Seq), dual RNA-Seq, microscopy, and bioassays were reared under controlled conditions at the Max Planck Institute for Biology (Tübingen, Germany): 26 °C, 60% relative humidity, and a 14.5-hour light/9.5-hour dark cycle, with continuous access to their host plant, *Ipomoea batatas*. Additional Cassidinae beetle species, including *Chelymorpha bullata*, *Chelymorpha gressoria*, and *Aspidimorpha quinquefasciata*, were maintained under identical conditions. *Charidotella sexpunctata* and *Acromis sparsa* were collected in Gamboa, Panamá from leaves of *Ipomoea batatas* and *Merremia umbellata*, respectively. *Cassida rubiginosa* was collected from *Cirsium oleraceum* in Tübingen, Germany.

### Genome sequencing and assembly

To generate the first draft genome of *Chelymorpha alternans*, high molecular weight (HMW) DNA was extracted from two adult females using the Monarch® HMW DNA Extraction Kit for Tissue (New England Biolabs, NEB). DNA integrity and fragment size were assessed using the Femto Pulse System (Agilent). HMW DNA was sheared to approximately 25 kb using Megaruptor 2 (Diagenode).

A high-fidelity (HiFi) sequencing library was prepared using the SMRTbell® Express Template Prep Kit 2.0 (PN 101-853-100; Pacific Biosciences). Library was size-selected using the BluePippin System (Sage Science), and appropriate fractions were verified on the Femto Pulse System. Pooled fractions were purified and concentrated using AMPure® PB beads (PacBio). Final library concentration was measured using the Qubit™ 1X dsDNA HS Assay Kit (Thermo Fisher Scientific), and size distribution was again confirmed on the Femto Pulse. Sequencing was conducted on two 8 M SMRT® Cells on the PacBio® Sequel II System (Pacific Biosciences) at the Max Planck Institute for Biology (Supplementary Data 4).

HiFi reads (quality >Q20) were generated using the circular consensus sequencing (CCS) analysis via the pbccs tool from the pbbioconda package (--min-passes 3 --min-rq 0.99 --min-length 10 --max-length 50000). CCS reads from both SMRT cells were merged and assembled into contigs using Hifiasm (v0.14.1-r314)^80^ with default parameters. The resulting contig-level assembly was filtered to remove heterozygous contigs using Redundans^81^. Contaminant sequences were identified and removed with BlobTools2^82^ and further deduplication was performed using the funannotate clean module from the Funannotate pipeline^83^. Genome assembly quality and completeness were assessed using BUSCO^84^ v5.1.2 with the OrthoDB v10 ^85^ Endopterygota gene set (2,124 conserved single-copy orthologs).

### Genome annotation

Genome annotation was conducted with the Funannotate pipeline^83^. Repetitive elements were identified and soft-masked via RepeatMasker (v4.0.7)^86^ and RepeatModeler (v2.0.3)^87^. Gene prediction combined ab initio models with transcriptomic evidence. RNA-Seq reads (NCBI SRA accession: SRR10030205) were aligned with HISAT2 (v2.2.1)^88^, assembled into transcript models by StringTie (v2.2.1)^89^, and refined through PASA (v2.5.2)^90^.

Ab initio gene models were generated with Augustus (3.3.3)^91^, SNAP (v2006-07-28)^92^, and GeneMark-ES (v4.69)^93^. Gene predictors were trained on PASA-filtered gene models, incorporating intron hints from RNA-Seq alignments to enhance accuracy. Final consensus gene models were generated with EvidenceModeler (v1.1.1)^94^ by integrating weighted input from gene predictors, protein alignments, and transcript evidence. Transfer RNAs were annotated with tRNAscan-SE (v2.0.9)^95^ in eukaryotic mode. Gene models were further refined to incorporate untranslated regions (UTRs) through the Funannotate ‘update’ module, which utilizes transcript abundance estimates from RNA-Seq data.

For functional annotation, protein sequences were analyzed using HMMER (v3.3.2)^96^ against the Pfam (v35.0) database^97^. Homology searches were conducted with DIAMOND (v2.0.15)^98^ against UniProt (release 2022_01)^99^ and MEROPS (v12.0)^100^. Carbohydrate-active enzymes (CAZymes) were identified with dbCAN (v10.0)^101^ HMMs. Orthology-based functional terms including Clusters of Orthologous Genes (COG) classifications, Gene Ontology terms, and KEGG pathway assignments, were obtained through eggNOG-mapper (v2.1.7)^102^ using the eggNOG v5.0 database. Protein product names were standardized using Gene2Product (v1.77), and candidate secreted proteins were predicted with SignalP (v5.0)^103^.

### Organ-specific RNA sequencing analysis

Transcriptomic profiles of the foregut symbiotic organs and ovary-associated glands in *C. alternans* were assessed by RNA sequencing. For each of three biological replicates, three reproductively adult females were sampled at 24 days post metamorphosis. Foregut symbiotic organs and ovary-associated glands were dissected from the same individual, and tissues were pooled per replicate.

Total RNA was extracted immediately after dissection using the QIAGEN RNeasy Mini Kit following Protocol 4: Enzymatic Lysis and Proteinase K Digestion of Bacteria (starting at step 7) from the RNAprotect® Bacteria Reagent Handbook (QIAGEN), as implemented in ref. ^34^. RNA concentration was quantified on a Qubit fluorometer (Thermo Fisher Scientific) with the Qubit™ RNA High Sensitivity (HS) Kit.

Ribosomal RNA was depleted from 30 ng of total RNA using the NEBNext rRNA Depletion Kit (Human/Mouse/Rat), followed by library construction with the NEBNext Ultra II Directional RNA Library Prep Kit (New England Biolabs). Library quality and fragment size were assessed on an Agilent 2100 Bioanalyzer with the High Sensitivity DNA Kit (Agilent Technologies). Libraries were sequenced on an Illumina HiSeq 3000 platform (paired-end 2 × 150 bp) at the Max Planck Institute for Biology (Tübingen, Germany), targeting a depth of ~30 million reads per library (Supplementary Data 4).

Raw reads were adapter-trimmed and quality-filtered with Trimmomatic (v0.36)^104^. Filtered reads were aligned to the *C. alternans* reference genome using HISAT2 (v2.2.0)^88^. Gene-level counts were obtained with HTSeq (v0.11.5)^105^ (stranded=reverse -r pos) using the *C. alternans* genome annotation file. Differential gene expression was analyzed in R (v4.3.1) with the DESeq2^106^ package, applying an adjusted False Discovery Rate (FDR) < 0.05 and a |log₂ fold-change | > 2 as significance thresholds. A volcano plot illustrating differentially expressed genes was created with the EnhancedVolcano^107^ R package.

### Protein separation by SDS-PAGE and identification by LC-MS

Symbiont-bearing spheres were isolated from 30 *C. alternans* eggs and homogenized in 20 μl of 6X SDS-PAGE loading buffer (Carl Roth). The lysate was denatured at 95 °C for 10 minutes and loaded onto a 10% SDS-PAGE gel. After electrophoretic separation, proteins were visualized using Coomassie InstantBlue® Protein Stain (Abcam). A predominant band migrating at ~120 kDa, larger than the predicted ~98 kDa molecular weight of Yellow, was excised and submitted for LC-MS analysis (TOPLAB GmbH (Martinsried, Germany). Protein identification was performed using peptide mass fingerprinting (PMF). Samples underwent reduction and alkylation of cysteines, followed by tryptic digestion. Peptides were desalted using ZipTip RP C18 and analyzed via LC-ESI-MS on a maXis plus mass spectrometer (Bruker) coupled to a Vanquish Flex UHPLC system (Thermo). Mass spectra were searched against the provided sequence database using Mascot software, confirming the identity of Yellow in *C. alternans*. To determine the sites of N-glycosylation, the sample was analyzed both with and without PNGase F treatment. The presence of deaminated peptides in the treated sample and their absence in the untreated sample served as indicators for N-glycosylation sites.

### Phylogenetic analysis of Yellow proteins

Putative *yellow* protein-coding genes in *C. alternans* were identified through the genome annotation pipeline described above. The corresponding amino acid sequences were extracted and included in a phylogenetic analysis alongside 269 Yellow amino acid sequences from hymenopteran, coleopteran, and dipteran insects, as well as six bacterial sequences. Sequences were aligned using MAFFT (v1.4.0)^108^ with default parameters. The best-fit substitution model was determined using ModelTest-NG (v0.2.0)^109^. A Maximum Likelihood (ML) phylogenetic tree was constructed using RAxML-NG (v1.2.0)^110^ (ngen=1000) under the selected model.

### Quantification of *yellow* expression in *C. alternans* body compartments by real-time qPCR

Expression of *yellow* was quantified across various body compartments in *C. alternans* using real-time qPCR (RT-qPCR). Across four biological replicates, one female adult was sampled for each replicate 1- and 31 days post-metamorphosis, and one male adult was sampled for each replicate 31 days post-metamorphosis. Female tissues included the foregut, foregut symbiotic organs, midgut, hindgut, ovaries, and ovary-associated glands. For males, foregut, foregut symbiotic organs, midgut, hindgut, and testes were dissected.

Total RNA was isolated from each tissue with the QIAGEN RNeasy Mini Kit, (including on-column DNase digestion. RNA quantity was assessed using a Qubit fluorometer (Thermo Fisher Scientific) with the Qubit™ RNA High Sensitivity (HS) Kit. For cDNA synthesis, 10 ng of total RNA per sample was reverse-transcribed using the RevertAid First Strand cDNA Synthesis Kit (Thermo Fisher Scientific) with oligo(dT) primers. Gene expression was quantified by real-time qPCR (RT-qPCR) on an Analytik Jena qTOWER³ cycler with the Platinum SYBR Green qPCR SuperMix-UDG (Thermo Fisher Scientific). Thermal cycling conditions were as follows: 95 °C for 10 min, followed by 45 cycles of 95 °C for 30 s and 60 °C for 20 s, with a final melting curve from 60 to 95 °C for 30 s. Standard curves were constructed from a 10-fold serial dilution of cDNA (10^−1^ to 10^−8^ ng/μl) for both *yellow* and the reference gene (eukaryotic elongation factor 2, *EF-2*). Gene-specific primers were designed and validated by Sanger sequencing of the amplicons (Table S5). Absolute transcript counts were interpolated from Ct values using the corresponding standard curves. For each sample, the expression value of *yellow* was first normalized to *EF-2*, then to the average expression measured in the ovary-associated glands of females 31 days following adult eclosion to obtain final fold-change values. After confirming that the data did not meet normality assumptions using the Shapiro-Wilk test, differences in *yellow* expression across female tissues were analyzed using a Kruskal-Wallis test, followed by Wilcoxon rank-sum tests for pairwise comparisons. Bonferroni correction was applied to adjust for multiple testing.

Because *yellow* expression was undetectable in all male tissues, a Wilcoxon rank-sum test was used to assess sex-specific differences in expression.

To assess age-related expression changes in the ovary-associated glands, a two-sided *t*-test was performed following confirmation of normality, comparing females 1 and 31 days following adult eclosion.

### Heterologous expression of *yellow* in *Escherichia coli* and antibody generation

The open reading frame corresponding to *yellow* exon 1 was synthesized by Eurofins Genomics and cloned into the pnEvH-His vector between the *BamHI* and *NdeI* restriction sites under the control of a T7 phage promoter (construct: CE7_Yellow-Protein-Exon1). Plasmid integrity was confirmed via Sanger sequencing.

For protein production, the verified construct was transformed into *E. coli* Gold pLysS cells and plated on LB agar containing kanamycin. Colonies from two plates were inoculated into 10 ml LB medium, then expanded into 1 L LB and cultured at 37 °C with shaking (180 rpm) until an OD_600_ of 0.4–0.6 was reached. The culture was cooled to 20 °C for 15 min, induced with 1 mM IPTG, and incubated overnight at 20 °C with continued shaking.

Cells were harvested by centrifugation (1717 x g, 20 min, 4 °C), resuspended in 20 ml of lysis buffer (20 mM Tris-HCl, pH 7.5, 1 M NaCl, 2 M urea, 50 mM imidazole, pH 8.0), and treated with DNase and lysozyme. Cell lysis was carried out by sonication, and the lysate was clarified by centrifugation (27,220 x g, 45 min, 4 °C). The supernatant was incubated with HisPur™ Cobalt Resin (Thermo Fisher Scientific) for 1 h at 4 °C and loaded onto a BioRad disposable chromatography column. The resin was washed three times with 4 column volumes (CV) of lysis buffer, and bound protein was eluted with 2 CV of elution buffer (20 mM Tris-HCl, pH 7.5, 1 M NaCl, 2 M urea, 200 mM imidazole, pH 8.0). The eluted was dialyzed overnight against 2 L of dialysis buffer (20 mM Tris-HCl, pH 7.5, 1 M NaCl, 2 M urea) using a 5 kDa molecular weight cutoff membrane (Spectra/Por). The protein was concentrated in a spin column and diluted to 1 mg/ml in storage buffer (20 mM Tris-HCl (pH 7.5), 0.2 M NaCl, 2 M urea) before sending to Davids Biotechnologie GmbH (Regensburg, Germany) for custom polyclonal antibody production in rabbit.

### Organ-specific RNA sequencing in additional tortoise beetle species

Ovary-associated glands were dissected from six reproductively active tortoise beetle species: *Acromis sparsa*, *Chelymorpha bullata*, *Chelymorpha gressoria*, *Charidotella sexpunctata*, *Cassida rubiginosa*, and *Aspidimorpha quinquefasciata*. Total RNA was extracted using the QIAGEN RNeasy Mini Kit following the manufacturer’s protocol, including on-column DNase digestion. RNA quantity was assessed with a Qubit fluorometer using the Qubit™ RNA Broad Range (BR) Kit (Thermo Fisher Scientific). Between 450 and 600 ng of total RNA per sample was used for library preparation. Poly(A) mRNA was isolated with the NEBNext® Poly(A) mRNA Magnetic Isolation Module (New England Biolabs), and RNA-Seq libraries were prepared using the NEBNext® Ultra™ II Directional RNA Library Prep Kit (New England Biolabs). Library fragment size was verified using an Agilent 2100 Bioanalyzer using the High Sensitivity DNA Kit (Agilent Technologies). Final libraries were sequenced on an Illumina NextSeq 2000 system (paired-end 2 × 100 bp) at the Max Planck Institute for Biology (Tübingen, Germany), yielding approximately 20-50 million reads per sample (Supplementary Data 4).

### Identification of Yellow proteins in additional tortoise beetle species

Raw reads were adapter-trimmed and quality-filtered using Trimmomatic (v0.36)^104^. De novo transcriptome assemblies were generated with Trinity (v2.8.5)^111^ using the parameters --normalize_by_read_set --SS_lib_type RF. Assemblies encompassed data from six newly sequenced cassidine species, as well as ovary-associated gland RNA-Seq data from *C. alternans*. Assembly completeness was evaluated with BUSCO (v5.1.2)^84^ using the Endopterygota gene set from OrthoDB (v10)^85^. Transcript abundance was quantified with RSEM^112^. For each species, the top 10,000 transcripts were used to generate box plots of normalized transcript counts expressed as transcripts per million (TPM). Protein-coding sequences were predicted with TransDecoder (v5.5.0)^113^ and annotated with HMMER (v3.3.2)^96^ against the Pfam database^97^. Sequences annotated as Yellow proteins were extracted from each species’ predicted proteome and compared to the *C. alternans* Yellow sequence using BLAST.

To confirm the identity of the candidate proteins, symbiont-bearing spheres were isolated from 30 to 60 eggs of each beetle species and homogenized in 20 μl of 6X SDS-PAGE loading buffer. Lysates were denatured at 95 °C for 10 min and loaded onto 10% SDS-polyacrylamide gels. Proteins were separated by electrophoresis and visualized using Coomassie InstantBlue® Protein Stain (Abcam).

Distinct gel bands were excised and submitted to the Quantitative Proteomics Facility at the University of Tübingen for analysis. Protein samples were subjected to in-gel digestion with sequencing-grade trypsin following reduction with dithiothreitol (DTT) and alkylation with iodoacetamide (IAA). Peptides were extracted, desalted using C18 StageTips, and analyzed by liquid chromatography-tandem mass spectrometry (LC-MS/MS) using an EASY-nLC 1200 system coupled to a Q Exactive HF mass spectrometer (Thermo Fisher Scientific). Peptides were separated using a 46-min gradient (10–50% acetonitrile in 0.1% formic acid) at a flow rate of 200 nl min⁻¹.

Raw MS/MS spectra were processed using MaxQuant^114^ (v1.5.2.8 and v2.4.2.0) with the Andromeda search engine and searched against a species-specific protein database derived from Trinity-assembled transcriptomes. Trypsin specificity allowing up to two missed cleavages, carbamidomethylation of cysteine as a fixed modification, and oxidation of methionine and N-terminal acetylation as variable modifications were used. Peptide and protein identifications were filtered at 1% false discovery rate using a target-decoy strategy. Protein quantification was performed using the iBAQ algorithm implemented in MaxQuant. This analysis confirmed the presence of the Yellow protein in *Acromis sparsa*, *Aspidimorpha quinquefasciata*, *Cassida rubiginosa*, *Chelymorpha bullata*, and *Chelymorpha gressoria*.

Mass spectrometry proteomics data have been deposited to the ProteomeXchange Consortium via the PRIDE partner repository with the dataset identifier PXD075214.

### Selection analyses

Codon-level selection on the *yellow* gene was evaluated using the Fixed Effects Likelihood method^53^ implemented on the Datamonkey web server (https://www.datamonkey.org)^115^. Protein sequences from various tortoise beetle species were aligned with MUSCLE (v5.1)^116^, and a codon-aware nucleotide alignment was generated with PAL2NAL (v14)^117^. A Maximum Likelihood phylogeny was inferred automatically by Datamonkey and used alongside the codon alignment as input for the FEL analysis. Codon sites were considered to be under selection when the nonsynonymous substitution rate (d*N*) significantly differed from the synonymous rate (d*S*) at *p* < 0.1. The analysis identified 234 codon sites under purifying (negative) selection (d*N* < d*S*) and 8 sites under diversifying (positive) selection (d*N* > d*S*). To test for enrichment of positively selected sites within specific regions of *yellow*, the number of such sites in Exon 1 (7 sites) was compared to those in Exons 2 and 3 (1 site) using a Fisher’s exact test. Exons 2 and 3 were grouped to represent the C-terminal region of the *yellow* gene. Region lengths were based on codon positions from the FEL alignment (346 codons in Exon 1; 340 codons in Exons 2 and 3). A second Fisher’s exact test was applied to assess whether Exon 1 (69 sites) was significantly depleted of codon sites under purifying selection compared to Exons 2 and 3 (165 sites).

### Structural modeling of Cassidinae Yellow proteins and functional site mapping

Candidate Yellow sequences were submitted to the AlphaFold3 server^118^ (https://alphafoldserver.com/) for structure prediction. Structural alignment of the predicted models was performed using the align function in PyMOL^119^, with *C. alternans* Yellow selected as the reference (FUN_023368 from GCA_056790185.1). A custom PyMOL script was used to calculate per-residue RMSD values by measuring distances between Cα atoms across the aligned models. RMSD values were stored in the B-factor field of the reference model and visualized using a red-blue color gradient, where red indicates low structural variability and low indicates high variability.

Model confidence scores (pLDDT) for each protein were extracted from AlphaFold predictions and summarized by exon region (Table S4). Exon boundaries were defined based on the *C. alternans* Yellow gene model. For the remaining species, exon 1 and exons 2-3 were inferred by aligning transcript-derived sequences to the *C. alternans* reference due to the absence of annotated exon structures.

To investigate putative functional sites, hydrophobic residues were identified in PyMOL^119^, and highlighted in yellow based on amino acid identity (Ala, Val, Leu, Ile, Met, Phe, Trp, and Pro). Positively selected sites, identified through molecular evolution analysis, were mapped onto the predicted protein structure and visualized in red. This approach allowed the spatial relationship between conserved hydrophobic patches and evolutionary hotspots to be assessed.

### Symbiont-Yellow cophylogenetic analysis

Yellow protein sequences from tortoise beetle species were aligned using MUSCLE (v5.1)^116^, including *Leptinotarsa decemlineata* (Coleoptera: Chrysomelidae: Chrysomelinae) as an outgroup. The optimal substitution model for the alignment was determined using ModelTest-NG (v0.2.0)^109^. For the symbiont phylogeny, 61 previously identified core symbiont protein sequences^34^, along with sequences from four outgroup taxa (*Buchnera aphidicola*, *Escherichia coli*, *Vibrio fisheri*, and *Candidatus* Ishikawaella capsulata), were concatenated and aligned using MUSCLE (v5.1)^116^.

PartitionFinder (v2.1.1)^120^ was used to determine the best-fit substitution model for each partition with parameters set to branchlengths = unlinked, models = GTR, GTR + G, GTR + I + G, model_selection = bic. Maximum Likelihood (ML) phylogenies were inferred using RAxML-NG (v1.2.0)^110^ (ngen=1000) under the respective best-fit models.

The resulting trees were visualized side-by-side in Dendroscope3^121^ as a tanglegram using the Neighbor Net Tanglegram algorithm. Cophylogenetic congruence between *yellow* genes and *Stammera* symbionts was assessed using eMPRess GUI^122^. Equal costs (1) were assigned to duplication, transfer, and loss events. Significance was evaluated by comparing the observed reconciliation cost to a null distribution generated through randomized tip associations (*p* < 0.01).

### Expression of *yellow* in ovary-associated gland segments and sphere-storing organs

To quantify *yellow* expression across ovary-associated gland segments and sphere-storing organs, three biological replicates were analyzed. For each replicate, a single adult female was sampled at day 1 and 31 following adult eclosion. Segments 1-3 of the ovary-associated glands, as well as the sphere-storing organs were dissected from the same individual. Total RNA was isolated using the QIAGEN RNeasy Mini Kit, including on-column DNase digestion. RNA concentration was quantified with a Qubit fluorometer (Thermo Fisher Scientific), and 7 ng of RNA were reverse-transcribed into cDNA using the RevertAid First Strand cDNA Synthesis Kit (Thermo Fisher Scientific) using oligo (dT) primers.

Quantitative real-time PCR (RT-qPCR) was carried out on an Analytik Jena qTOWER³ using the Platinum SYBR Green qPCR SuperMix-UDG (Thermo Fisher Scientific). Cycling conditions were as described previously. Absolute expression levels of *yellow* were calculated from standard curves and normalized to those of the reference gene *EF-2*. For each ovary-associated gland segment, relative expression was determined by further normalizing this ratio to that of segment 3, yielding fold-change values.

Log transformation was applied to data from 1-day old females, while square root transformation was used for 31-day old females. After confirming assumptions of normality and homoscedasticity by Shapiro and Levene’s tests, respectively, general linear models (LMs) were fitted separately for each dataset, with segment and replicate included as fixed factors. Post hoc pairwise comparisons between gland segments were conducted using Tukey’s HSD test, implemented via the’glht’ function, with Bonferroni correction for multiple testing.

To compare *yellow* expression between ovary-associated glands and sphere-storing organs in 31-day old females, *yellow*/*EF-2* ratios were averaged across gland segments to obtain a single expression value per organ. These were then normalized to the mean expression in the sphere-storing organs, yielding fold-change values. Data were log-transformed to meet normality assumptions, and differences between the two organs were assessed using a two-sided *t*-test.

### Symbiont titers in ovary-associated gland segments and sphere-storing organs

Symbiont abundance was quantified in ovary-associated gland segments (segments 1-3) and sphere-storing organs across three biological replicates. For each replicate, one adult female was sampled at days 1 and 31 following adult eclosion. Total DNA was extracted using the QIAGEN Blood and Tissue Kit, including RNase treatment, following the manufacturer’s protocol. Quantitative PCR (qPCR) was carried out on an Analytik Jena qTOWER³ cycler using Platinum SYBR Green qPCR SuperMix-UDG (Thermo Fisher Scientific) under the following cycling conditions: 95 °C for 10 min, followed by 45 cycles of 95 °C for 30 s and 62.7 °C for 20 s, with a final melting curve from 60 °C to 95 °C for 30 s. Symbiont titers were estimated by amplifying the *groL* gene, and absolute copy numbers were interpolated from a standard curve generated using a 10-fold dilution series (10^−1^ to 10^−8^ ng/µl) of purified *groL* PCR product.

To assess differences in symbiont titers across ovary-associated gland segments in 1- and 31-day old females. General linear models were fitted separately for each dataset with segment and replicate as fixed factors following Box-Cox transformation and subsequent confirmation of normality and homoscedasticity. Post hoc pairwise comparisons between segments were conducted using Tukey’s HSD implemented via the’glht’ function, with Bonferroni correction for multiple testing.

*Stammera* titers in sphere-storing organs of females 1 and 31 days following adult eclosion were also compared. Data were log-transformed and differences between timepoints were assessed using a two-sided *t*-test after validation of a normal distribution.

### Double-stranded RNA (dsRNA) preparation

Total RNA was extracted from 31-day old, reproductively active *C. alternans* females using the QIAGEN RNeasy Mini Kit, including on-column DNase digestion. cDNA was synthesized from 10 ng of RNA using the First Strand cDNA Synthesis Kit (Thermo Fisher Scientific) with oligo(dT) primers. This cDNA served as template to amplify a 688 bp fragment corresponding to *yellow* exon 1 using Q5 High-Fidelity DNA Polymerase (New England Biolabs).

A 651 bp GFP control fragment was amplified from a laboratory plasmid containing superfolder GFP (sfGFP) with a C-terminal His tag in a pET expression vector backbone (pET30) originally derived from the superfolder GFP construct described by ref. ^123^. The sfGFP gene was cloned via NdeI/XhoI restriction sites and was used as a non-targeting control for RNA interference experiments. To avoid off-target knockdown effects, both sequences and primer pairs were aligned to the *C. alternans* genome using BLAST. Gene-specific primer sequences (Table S6) were verified by Sanger sequencing.

Gene-specific amplification including the T7 promoter was carried out from 2.5 μl cDNA using Q5® High-Fidelity PCR Kit (New England Biolabs) in two 50 μl reactions per gene (program for *yellow*: 98 °C 30 s, 5 x (98 °C 10 s, 63 °C 30 s, 72 °C 30 s), 30 x (98 °C 10 s, 68 °C 30 s, 72 °C 30 s), 72 °C 2 min, 4 °C on hold), (program for *GFP*: 98 °C 30 s, 5 x (98 °C 10 s, 60 °C 30 s, 72 °C 30 s), 30 x (98 °C 10 s, 65 °C 30 s, 72 °C 30 s), 72 °C 2 minutes, 4 °C on hold). Amplicons were confirmed by agarose gel electrophoresis and purified with the NucleoSpin® Gel and PCR Clean-Up Kit (Macherey-Nagel). One microgram of purified PCR product was used as template for double-stranded RNA synthesis with the MEGAscript™ T7 Transcription Kit (Thermo Fisher Scientific).

### RNA interference (RNAi) by dsRNA injection

Approximately 2 μg of dsRNA (targeting either *yellow* or *GFP*) was injected into the dorsal aperture between the head and thorax of ice-chilled, 31-day old female *C. alternans*. Across three replicates, five females were injected using a Microinjector FemtoJet® (Eppendorf) with pulled borosilicate glass capillaries (outer diameter: 1 mm, inner diameter: 15 μm) (BioMedical Instruments). A group of non-injected females served as an additional negative control. Following a brief recovery, females were placed in mesh cages (30 × 30 × 30 cm) containing a host plant and one male, and maintained under standard rearing conditions.

### Validation of RNAi-mediated *yellow* knockdown

To assess the effectiveness of RNAi, *yellow* expression levels were measured for five weeks following injection. At each time point (7-, 14-, 21-, and 35-days post-injection), one female per condition (dsRNA *GFP* and dsRNA *yellow*) was dissected, and ovary-associated glands were collected. Total RNA was extracted with the RNeasy Mini Kit (QIAGEN) with on-column DNase digestion. RNA concentration was measured with a Qubit fluorometer, and 10 ng RNA was reverse-transcribed using the RevertAid First Strand cDNA Synthesis Kit (Thermo Fisher Scientific) with oligo(dT) primers.

Expression of *yellow* was quantified via real-time PCR (RT-qPCR) using the Platinum SYBR Green qPCR SuperMix-UDG (Thermo Fisher Scientific) on an Analytik Jena qTOWER³ cycler. Cycling conditions were as described above. Absolute expression values were determined from standard curves and normalized to the reference gene *EF-2*. Ratios were then normalized to the average *yellow* expression measured in the ovary-associated glands of untreated females at each timepoint, producing fold-change values. Log transformed data were analyzed using general linear models (LMs) for each time point after verification that model assumptions were respected with condition and replicate included as fixed factors. Post hoc pairwise comparisons between treatment groups (untreated control, dsRNA *GFP*, and dsRNA *yellow*) were conducted using Tukey’s HSD implemented via the’glht’ function, with Bonferroni correction for multiple testing.

### Impact of *yellow* RNAi on symbiont titers under low-humidity conditions

To investigate whether *Stammera* is more susceptible to environmental stress during transmission in the absence of Yellow, three ice-chilled 31-day old *C. alternans* adult females per replicate and per condition were injected with 2 μg of dsRNA targeting wither *GFP* or *yellow*. Following injection, each female was individually paired with a male and housed in a cage (12 × 12 x 20) containing a host plant.

Eggs from untreated control, dsRNA *GFP* and dsRNA *yellow*-injected females were collected during the first two weeks post-injection. Collected eggs were then divided between two environmental conditions: a control condition (26 °C, 60% relative humidity) and low-humidity condition (26 °C, 45% relative humidity). After 1 and 9 days of incubation, one egg per treatment and per timepoint was collected for co-extraction of DNA and RNA using the InnuPREP DNA/RNA Mini Kit (IST Innuscreen GmbH), including lysozyme pretreatment.

Quantification of symbiont titers was performed by qPCR targeting the *groL* gene, using Platinum SYBR Green qPCR SuperMix-UDG (Thermo Fisher Scientific) on an Analytik Jena qTOWER^3^ .Cycling conditions were as described above. Standard curves were generated from a 10-fold dilution series (10^−1^ to 10^−8^ ng/μl) of purified PCR products, allowing absolute gene copy number to be interpolated from the threshold cycle (Ct) values.

Statistical analyses were conducted separately for each collection day and treatment group. For eggs collected on day 9, symbiont copy numbers were modeled using a negative binomial generalized linear model (NB-GLM) with a log link function, implemented via the glm.nb() function in the MASS package. This model was chosen to account for overdispersion in the data, as evidenced by variance exceeding the mean, and was further supported by lower AIC values compared to alternative distributions.

### *Stammera* transcriptional response to low humidity stress in the absence of Yellow

To investigate symbiont gene expression across environmental conditions and treatment groups, RNA was quantified using a Qubit fluorometer (Thermo Fisher Scientific) with the Qubit™ RNA High Sensitivity (HS) Kit. A total of 18 ribosome-depleted RNA libraries were prepared from 55 ng of total RNA using the NEBNext rRNA Depletion Kit (Human/Mouse/Rat) and the NEBNext Ultra II Directional RNA Library Prep Kit (New England Biolabs). Library quality and fragment size distribution were assessed on an Agilent 2100 Bioanalyzer using the High Sensitivity DNA Kit (Agilent Technologies). Sequencing was performed on an Illumina NextSeq 2000 platform (paired-end 2 × 150 bp) at the Max Planck Institute for Biology (Tübingen, Germany), targeting a depth of approximately 20 million reads per library (Supplementary Data 4).

Raw reads were adapter-trimmed and quality-filtered using Trimmomatic (v0.36)^104^. Filtered forward reads were aligned to the *Stammera* genome using Bowtie2 (v2.3.5.1)^124^ (--fr, --no-unal). Gene counts were summarized with featureCounts^125^ (-s 2) as part of the Subread package release 2.0.0.

Differential gene expression was assessed independently within each treatment group (untreated control, dsRNA *GFP* and dsRNA *yellow*) across environmental conditions (control and low-humidity) using the DESeq2^106^ package in R (v4.3.1), applying an adjusted False Discovery Rate (FDR) < 0.05 and a log_2_ fold change > 1 as significance thresholds.

To account for variability in bacterial biomass across samples, bacterial titers measured by qPCR were included as a covariate in the DESeq2 design formula ( ~ Bacterial_titer + Replicate + Treatment). Incorporating titer values into the differential expression model helped correct for global transcriptional shifts associated with differences in bacterial load, ensuring that observed changes in expression were attributed to dehydration responses, rather than variation in total bacterial RNA content. While DESeq2 accounts for library size differences through size factor normalization, this can lead to inflated fold-change estimates in samples with very low counts, including bacterial titers mitigated this issue.

The number of genes with log_2_ fold-change <–1 or > 1 in untreated control and dsRNA *GFP* conditions were compared to those in the dsRNA *yellow* using a Fisher’s exact test.

Volcano plots were generated for each treatment group across environmental conditions using the EnhancedVolcano^107^ R package. Heatmaps of the log(x + 1)-transformed normalized read counts were constructed using the pheatmap package^126^ to visualize expression patterns across samples.

### Preparation of paraffin sections

Foregut symbiotic organs, ovary-associated glands, sphere-storing organs and eggs were fixed in Carnoy’s solution (ethanol:chloroform:acetic acid, 6:3:1) at room temperature overnight. Fixed tissues were dehydrated in a graded ethanol series: 70 and 80% (10 min each), followed by 96% and 100% (10 min each, three times). Ethanol was replaced by Roti®-Histol at room temperature (2 × 40 min, then overnight). Samples were subsequently incubated at 60 °C for 1 h in a 1:1 mixture of Roti-Histol and paraffin, and then transferred to pure paraffin at 60 °C (3 ×1 h, 1 x overnight).

Paraffin-embedded samples were sectioned at 10 μm using a conventional microtome and mounted on poly-L-lysine-coated glass slides (Epredia, Germany) using a 40 °C water bath. Sections were air-dried overnight at room temperature and incubated at 60 °C in vertical position for 1 h to improve tissue adherence.

### Fluorescence in situ hybridization (FISH) on paraffin sections

Paraffin Sections were dewaxed in Roti®-Histol (3 × 10 min), followed by immersion in 96% ethanol for 10 min at room temperature. Slides were dried at 37 °C for 30 min. Hybridization of ovary-associated glands, sphere-storing organs and eggs was performed using the SAL227-Cy5 probe, which targets the 16S rRNA sequence of *Stammera* from *C. alternans* (5’GGTCTTGAAAAAAAAAGATCCCC’3)^34^. The foregut symbiotic organ sample of Fig. 1D was hybridized with the SCA600-Cy5 probe, which targets the 16S rRNA sequence of *Stammera* from 52 tortoise beetle species (5’AAACCACCTACATGCTCTTTACGCCC’3)^34^. Probes were dissolved at 900 nM in the hybridization buffer [35% formamide (v/v), 900 mM NaCl, 20 mM Tris-HCl pH 7.8, 1% nucleic acid blocking reagent (v/v), 0.02% SDS (v/v), 10% dextran sulfate (w/v)].

Hybridization was carried out at 46 °C for 4 h. Sections were rinsed in 48 °C washing buffer [70 mM NaCl, 20 mM Tris-HCl pH 7.8, 5 mM EDTA pH 8.0, and 0.01% SDS (v/v)], then transferred to fresh 48 °C washing buffer for 15 minutes. Slides were washed in phosphate-buffered saline (PBS) (20 min) and milliQ water (1 min) at room temperature. Sections were counterstained with DAPI (5 μg/ml) for 10 min at room temperature, followed by milliQ water and 100% ethanol rinses. After drying at 30 °C for 30 min, slides were mounted using ProLong® Gold Antifade Mountant (Thermo Fisher Scientific), cured overnight at room temperature, and visualized using an LSM 980 NLO confocal microscope (Zeiss, Germany).

### Immunohistochemistry on paraffin sections

Paraffin sections were dewaxed in Roti®-Histol (3 × 10 min) and rehydrated in an ethanol gradient series (96% for 10 min, 80 and 60% for 5 min each). The deparaffinized sections were washed in distilled water (3 × 3 min) and incubated in blocking buffer (3% bovine serum albumin in PBS, 0.5 % Tween-20) for 2 hours at 4 °C. Slides were incubated in a humidified chamber and incubated with the custom-made primary antibody (1:10 anti-Yellow) raised in rabbit (1.4 mg/ml, 0.02% sodium azide) in blocking buffer for 16 h at 4 °C. After rinsing the sections with 1x PBS (3 × 3 min), samples were incubated with the secondary antibody (Alexa Fluor 568-conjugated goat anti-rabbit IgG (2 mg/ml; Thermo Fisher Scientific; catalog number A-110011; lot number 2088068)) (1:200) in blocking buffer for 1 h in a humidified chamber at room temperature. Sections were rinsed again using 1x PBS (3 × 3 min).

Sections were counterstained with DAPI/PBS (5 μg/ml) for 10 min at room temperature, dipped in milliQ water and dipped in ethanol 100%. Slides were mounted using the ProLong® Gold antifade mounting media (Thermo Fisher Scientific), cured overnight at room temperature, and visualized using an LSM 980 NLO confocal microscope (Zeiss, Germany).

### Whole-mount fluorescence in situ hybridization (FISH)

Whole-mount FISH was performed on the *C. alternans* digestive tract, ovary-associated glands and sphere-storing organs. After fixation in Carnoy’s solution overnight at room temperature, tissues were incubated in 6% H_2_O_2,_ 80% ethanol solution to bleach tissue pigmentation. Samples were then washed with 70% ethanol and rehydrated by three 2-minute washes in PBSTx (0.8% NaCl, 0.02% KCl, 0.115% Na₂HPO₄, 0.02% KH₂PO₄, 0.3% Triton X-100), followed by three 2-minute washes in freshly prepared hybridization buffer (20 mM Tris-HCl pH8, 0.9 M NaCl, 0.01% SDS, 30% formamide). Hybridization was carried out overnight at room temperature (24 °C) using probes at a final concentration of 100 nM and DAPI at 100 ng/ml in hybridization buffer. The next day, tissues were washed three times for 2 minutes in PBSTx, mounted using the ProLong® Gold antifade mounting media (Thermo Fisher Scientific) and imaged with a LSM 980 NLO confocal microscope (Zeiss, Germany).

Hybridization of the ovary-associated glands and sphere-storing organs was performed using the SAL227-Cy5 probe, while the digestive tract sample was hybridized with a probe of identical sequence labeled with Atto550. All probes were dually labeled with fluorescent dyes at the 5’ and 3’ ends.

### Scanning electron microscopy of egg caplets

Egg caplets from *C. alternans* were fixed in 2.5% glutaraldehyde in PBS and post-fixed with 1% osmium tetroxide for 1 h on ice. Samples were washed and dehydrated through a graded ethanol series, with 24-hour incubations at each step, followed by critical point drying (CPD300, Leica). Egg caplets were then mounted on stubs and sputter-coated with a 10 nm layer of platinum (CCU-010, Safematic). Samples were examined using a field emission scanning electron microscope (Regulus 8230, Hitachi High Technologies) at an accelerating voltage of 3 kV.

### Ultrastructural and immunofluorescence/immunoelectron microscopy of egg caplets and sphere-storing organs

Eggs from *C. alternans* at two days post-oviposition were used to isolate symbiont-containing spheres for ultrastructural analysis. Approximately 25 egg caplets were carefully detached and mounted onto double-sided adhesive tape. The distal tips of the caplets, containing the symbiont-bearing spheres, were excised just beneath the chorionic membrane without disrupting it. Excised caplet tips were fixed in a solution containing 4% formaldehyde and 2.5% glutaraldehyde in PBS for 2 h at room temperature with gentle agitation on a rocking platform. After fixation, the specimens, which floated in the fixative, were collected using a fine mesh and transferred into 1.5 ml microcentrifuge tubes. Samples were subsequently incubated overnight at 4 °C.

*C. alternans* reproductively active adults were anesthetized using carbon dioxide (CO₂), and paired sphere-storing organs were dissected in phosphate-buffered saline (PBS) at room temperature (RT). Dissected tissues were immediately fixed in 4% formaldehyde in PBS for 2 h at RT, followed by overnight incubation at 4 °C.

Samples for ultrastructural analyses were post-fixed with 1% osmium tetroxide for 1 h on ice and 1% uranyl acetate for 1 h on ice, dehydrated in ethanol and embedded in Epon. Ultrathin sections were stained with uranyl acetate and lead citrate and analyzed with a Tecnai Spirit (Thermo Fisher Scientific) operated at 120 kV.

Samples for immunostaining were washed once in PBS and rinsed three times in distilled water. Post-fixation staining was performed in 1% aqueous uranyl acetate for 1 hour at 4 °C, followed by two rinses in distilled water. Dehydration was carried out at RT in a graded ethanol series: 30, 50, and 70% ethanol (30 min each), followed by two incubations in 100% ethanol (30 min each). Samples were then infiltrated with LR White resin via successive 1-hour incubations in 1:1 and 2:1 mixtures of LR White and ethanol, respectively. This was followed by overnight incubation in 100% LR White and two additional 4-hour incubations in fresh 100% LR White. Samples were transferred to gelatin capsules (11 blocks total) containing fresh LR White and polymerized at 48 °C for 3 days. Ultrathin sections were prepared at 50 nm thickness on 150 mesh copper grids for immunoelectron microscopy (IEM), or at 200 nm thickness on glass coverslips for immunofluorescence microscopy (IFM).

Sections were incubated in PBG blocking buffer (0.2% gelatin dissolved in PBS, cooled to RT, then supplemented with 0.5% BSA) for 10 min at RT. This was followed by incubation with primary antibody (rabbit anti-Yellow, 1.4 mg/ml, containing 0.02% sodium azide) diluted 1:20 in PBG for 1 h at 4 °C. Following primary antibody incubation, sections were washed six times for 3 min each in PBG. Secondary detection was performed using either Alexa Fluor 488-conjugated anti-rabbit IgG (Thermo Fisher Scientific; catalog number A-11008; lot number 6682-1) (dilution 1:500) for IFM, or a 6 nm Colloidal Gold-AffiniPure Goat Anti-Rabbit IgG (Jackson ImmunoResearch Labs; code number 111-195-144; lot number 102530) (1:20) for IEM, diluted in PBG and incubated for 1 hour and 30 minutes at RT.

Post-labeling washes included three rinses (3 minutes each) in PBG, three rinses in PBS, and four rinses in distilled water. For contrast enhancement, sections were incubated in 1% uranyl acetate in distilled water for 5 min, followed by four rinses in distilled water. IFM sections were mounted in Roti-Mount containing DAPI for nuclear counterstaining and visualized on a Zeiss Axioplan microscope. IEM sections were analyzed with a Tecnai Spirit (Thermo Fisher Scientific) operated at 120 kV.

### Statistical analyses

Statistical analyses, including Fisher’s exact test, Kruskal-Wallis test, Wilcoxon rank-sum tests, *t*-tests, ANOVA, and post hoc comparisons using Tukey’s HSD, were used to analyze selection signatures, gene expression across tissues, organs, and segments of the ovary-associated glands, RNAi knockdowns, and symbiont titers in organs and eggs. The specific statistical tests used for each analysis are detailed in the Methods section, figure legends, and in Supplementary Data 5. Multiple testing corrections were applied using either Bonferroni correction or False Discovery Rate (FDR) adjustment. A significance threshold of α = 0.05 was used for all tests. All statistical analyses were conducted in R (v4.3.1).

### Reporting summary

Further information on research design is available in the Nature Portfolio Reporting Summary linked to this article.

## Supplementary information

## Source data

## Data Availability

Genomic and transcriptomic sequencing data generated in this study have been deposited at the National Center for Biotechnology Information (NCBI) and are publicly available as of the date of publication under the BioProjects PRJNA1241163 and PRJNA1257053. The genome assembly generated in this study is deposited under the accession number GCA_056790185.1 [https://www.ncbi.nlm.nih.gov/datasets/genome/GCA_056790185.1/] on NCBI. SRA accession numbers for each dataset used/generated in this study can be found in Supplementary Data 4. Mass spectrometry proteomics data have been deposited to the ProteomeXchange Consortium via the PRIDE partner repository with the dataset identifier PXD075214. Raw data generated in this study have been deposited in the Zenodo repository https://doi.org/10.5281/zenodo.18746286 [https://zenodo.org/records/18746287]^127^. Further statistical analysis for Figs. 2–7 can be found in Supplementary Data 5. Source data are provided with this paper.

## Code availability

Code used for the analyses, including details of parameters and software, is available at https://github.com/mgarl-10/Yellow_protein [https://github.com/mgarl-10/Yellow_protein] and archived at Zenodo https://doi.org/10.5281/zenodo.18746286 [https://zenodo.org/records/18746287]^127^.
