# Supplementary material for: Yellow protein co-opted to sustain obligate symbiosis in leaf beetles
Source: Nat Commun. 2026 Sep 3;17:9446. doi: 10.1038/s41467-026-76942-1 (PMC13538373; doi:10.1038/s41467-026-76942-1)
Supplement: Supplementary file 1 [file 41467_2026_76942_MOESM1_ESM.pdf]

Supplementary information

Yellow protein co-opted to sustain obligate symbiosis in leaf beetles

Marleny García-Lozano[1], Christiane Emmerich[1,2,3], Christine Henzler[1], Iris Koch[4], Christa Lanz[5], Aftab Ayas[1], Inès Pons[1], Anja Buttstedt[6], Katharina Hipp[4], Hassan Salem[1,2,*]

[1] Mutualisms Research Group, Max Planck Institute for Biology, Tübingen 72076, Germany

[2] Department of Molecular Microbiology, John Innes Centre, Norwich NR4 7UH, United Kingdom

[3] The Sainsbury Laboratory, Norwich NR4 7UH, United Kingdom

[4] Electron microscopy facility, Max Planck Institute for Biology, Tübingen 72076, Germany

[5] Genome Centre, Max Planck Institute for Biology, Tübingen 72076, Germany

[6] Institute for Evolution and Ecology, University of Tübingen, Tübingen 72076, Germany

[*] For correspondence: hassan.salem@jic.ac.uk

A

```
1   MLIKASYGNY DQQTRDAQIN AQYARSSSGS SGSGRGYVDH VDYTAKINSA
51  NGGSHQFGYS GNDGKISTVH HFEAHGTQGS SENNHGSYLG NQQSSRARSR
101 YSRDQESSYI GHISERDGKI TGRYYYDQEH GSQGQHRFGY EHNNQHISGD
151 QSKRYRDNQE RRGTYYEVHD DVNQSGNQHR HEHDVRGFQV QSNSRDQGQS
201 NTAEYNGRND KITGRYQGVK GQSRSGNDNT RISGSRSRQS EHNENSGKIS
251 RLHQENHETK GHNVRSVSHI SKGSQAGCGQ SSEASHSTNN RLENRRKVRS
301 DSESSGQSYH QNKETRFHNV HHSRDHSQNS ANGNDIGSRK TFVLKTGTSQ
351 QSSSAGAGNV HSSRNIGSST GSRYINTNKR NSDQSESSSG EVRYSASISS
401 NNVQVEAASH INENNRRTTT ARSRFLRTTS HHEQPTRRVL IVKETTEVVP
451 NSRTRIQNHQ GGNTIETGRR RSHLHREHSR GSVSSSGSSS CGKFQVAYQW
501 NYFNFTWTCP YKMQHMRYSP ADYIPLSIAI YNNYMYMSFP RLRNNARVTL
551 ARMPLNSPRV NPVFTPYPSW EMNNGVTCES FQDVTKIEID KNGIMWVLDG
601 RRLNPSVNCP AKLYLVDLKQ DGRIIRRYNI PEPFCPESGC LLNDIVVDGD
651 YAFISDLTAG DEGIIVYNRL RNRSWKTRAR GHSEVFHYRH DSCSPNLFKT
701 DTSRVVAWIA LAPPCTTRNR TFLYVPEEIP GAFGIDVDIL KSQETATSDL
751 SDHIYQMFSK NGSTGAAIYD SKSTLYVGLL SQKTIGIMKC GDTELSVCPR
801 SNLIRYPGTF TFDNQDGSLY MTEMSSMDLS TTGVRTQEVN FKILKMKTNT
851 KSYMYCDGRN
```

B

```
1   MLIKASYGNY DQQTRDAQIN AQYARSSSGS SGSGRGYVDH VDYTAKINSA
51  NGGSHQFGYS GNDGKISTVH HFEAHGTQGS SENNHGSYLG NQQSSRARSR
101 YSRDQESSYI GHISERDGKI TGRYYYDQEH GSQGQHRFGY EHNNQHISGD
151 QSKRYRDNQE RRGTYYEVHD DVNQSGNQHR HEHDVRGFQV QSNSRDQGQS
201 NTAEYNGRND KITGRYQGVK GQSRSGNDNT RISGSRSRQS EHNENSGKIS
251 RLHQENHETK GHNVRSVSHI SKGSQAGCGQ SSEASHSTNN RLENRRKVRS
301 DSESSGQSYH QNKETRFHNV HHSRDHSQNS ANGNDIGSRK TFVLKTGTSQ
351 QSSSAGAGNV HSSRNIGSST GSRYINTNKR NSDQSESSSG EVRYSASISS
401 NNVQVEAASH INENNRRTTT ARSRFLRTTS HHEQPTRRVL IVKETTEVVP
451 NSRTRIQNHQ GGNTIETGRR RSHLHREHSR GSVSSSGSSS CGKFQVAYQW
501 NYFNFTWTCP YKMQHMRYSP ADYIPLSIAI YNNYMYMSFP RLRNNARVTL
551 ARMPLNSPRV NPVFTPYPSW EMNNGVTCES FQDVTKIEID KNGIMWVLDG
601 RRLNPSVNCP AKLYLVDLKQ DGRIIRRYNI PEPFCPESGC LLNDIVVDGD
651 YAFISDLTAG DEGIIVYNRL RNRSWKTRAR GHSEVFHYRH DSCSPNLFKT
701 DTSRVVAWIA LAPPCTTRNR TFLYVPEEIP GAFGIDVDIL KSQETATSDL
751 SDHIYQMFSK NGSTGAAIYD SKSTLYVGLL SQKTIGIMKC GDTELSVCPR
801 SNLIRYPGTF TFDNQDGSLY MTEMSSMDLS TTGVRTQEVN FKILKMKTNT
851 KSYMYCDGRN
```

Figure S1. LC-MS analysis of the Yellow protein of *Chelymorpha alternans* and identification of the N-glycosylation site. (**A**) Mascot database search results for the trypsin-digested sample. Peptide masses were searched against the customer-provided sequence and identified the Yellow protein of *Chelymorpha alternans* with a Mascot score of 7831 and 41% sequence coverage. Identified peptides are highlighted in bold red. The N-terminal region encoded by exon 1 is outlined in blue. (**B**) A parallel search identified the same protein with a score of 9636 and 45% coverage. The sample was analyzed with and without PNGase F treatment to determine the N-glycosylation site. Peptides 138–154 and 761–772 were found only after PNGase F treatment; only 761–772 showed deamidation, indicating N761 as the likely glycosylation site. Identified peptides are highlighted in bold red. The N-terminal region encoded by exon 1 is outlined in blue.

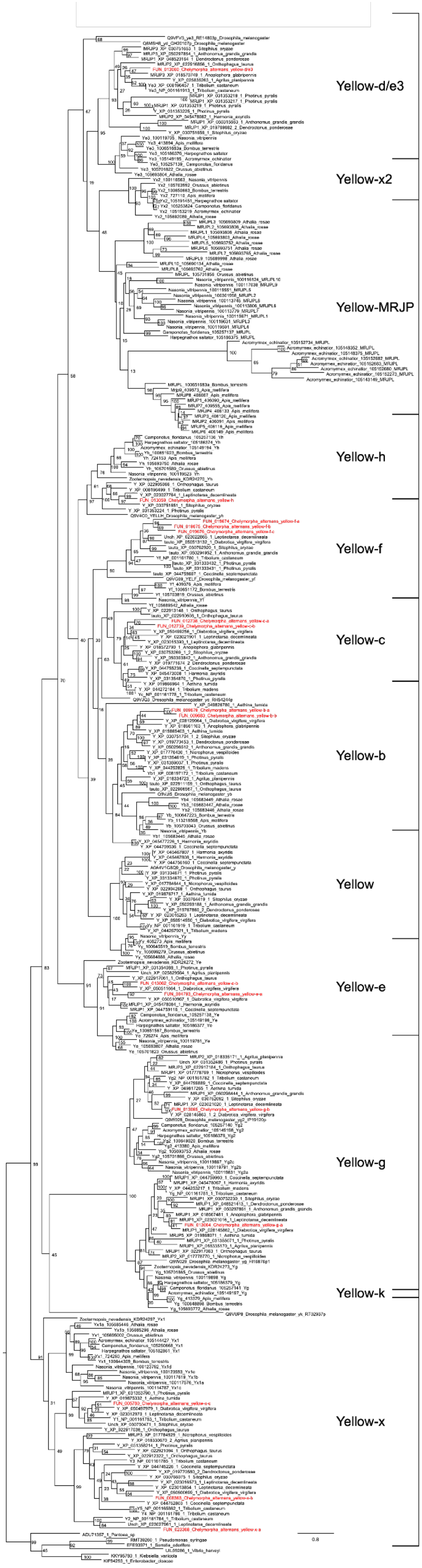

Figure S2. Extended version of the maximum likelihood phylogeny of 269 Yellow proteins from diverse insect taxa. The tree was constructed using RAxML under the LG+G4 substitution model. Labels on the right represent the classes to which the Yellow proteins belong. The scale bar indicates the number of amino acid substitutions per site. Yellow proteins from *Chelymorpha alternans* are indicated in red. Related to Figure 2.

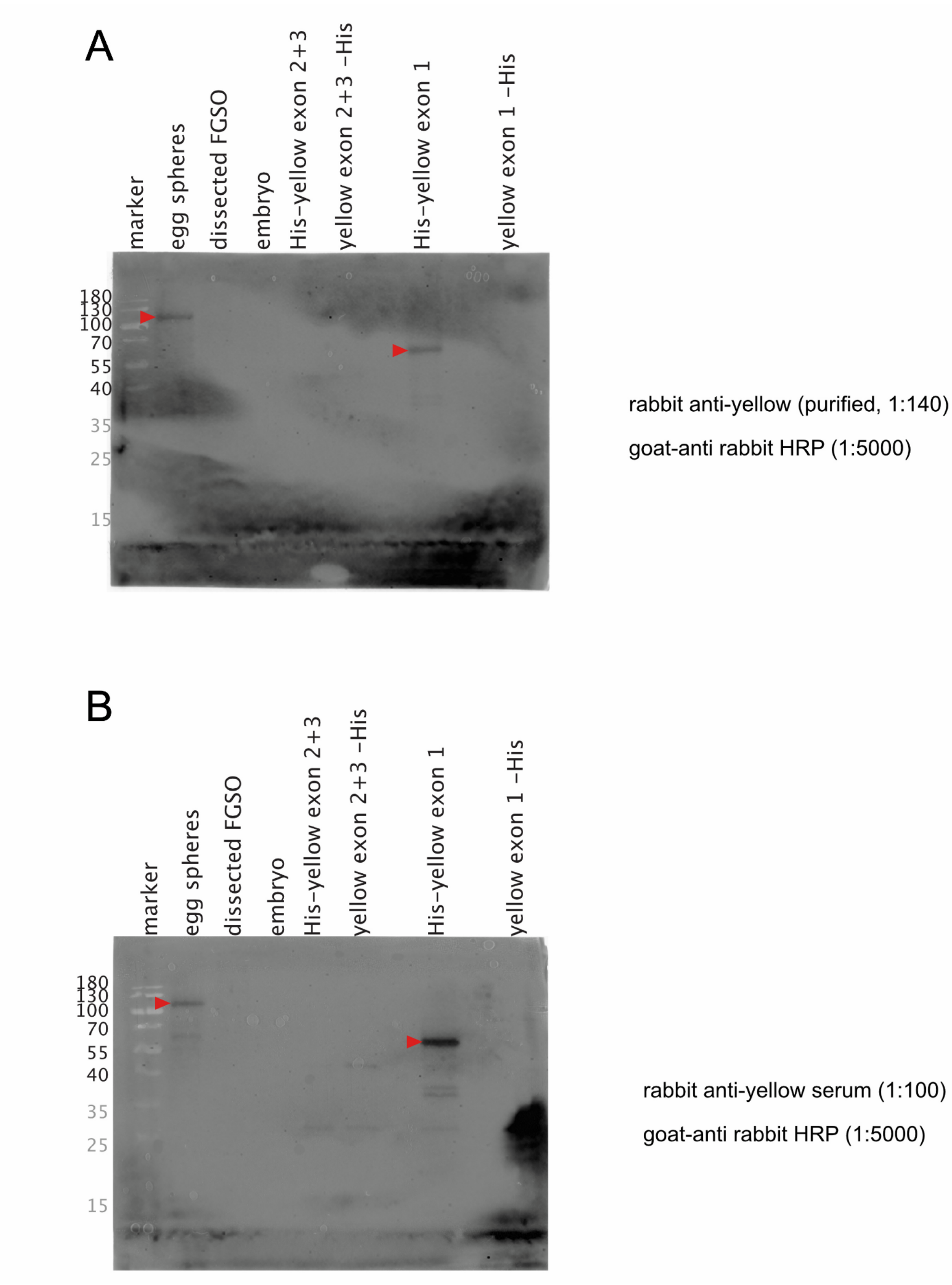

Figure S3. Western blots using the Yellow antibody to assess its specificity to exon 1. Western blot analysis shows a clear band in both the purified antibody (**A**) and serum (**B**) when tested on egg spheres and purified His-tagged Yellow exon 1 protein. No binding was observed in foregut symbiotic organs (FGSO), embryos, or in samples expressing only exons 2–3 of Yellow. The marker is in kilodaltons (kDa). This experiment has been repeated independently at least 3 times.

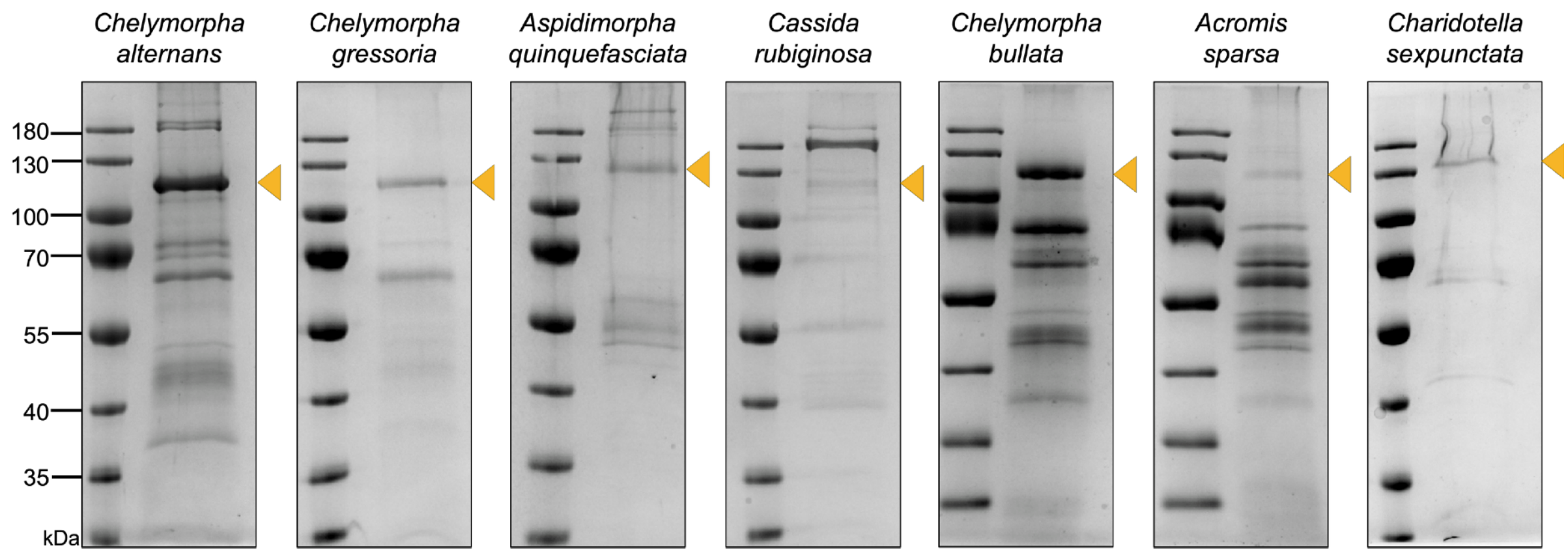

Figure S4. Sodium dodecyl sulfate-polyacrylamide gel electrophoresis (SDS-PAGE) of total proteins extracted from symbiont-bearing spheres isolated from eggs of various Cassidinae species. *Chelymorpha alternans* was used as a reference. The Yellow protein band is indicated by a yellow triangle. Protein identities were confirmed by LC-MS. Related to Figure 3. Experiments have been repeated independently at least 3 times. Source data are provided as a Source Data file.

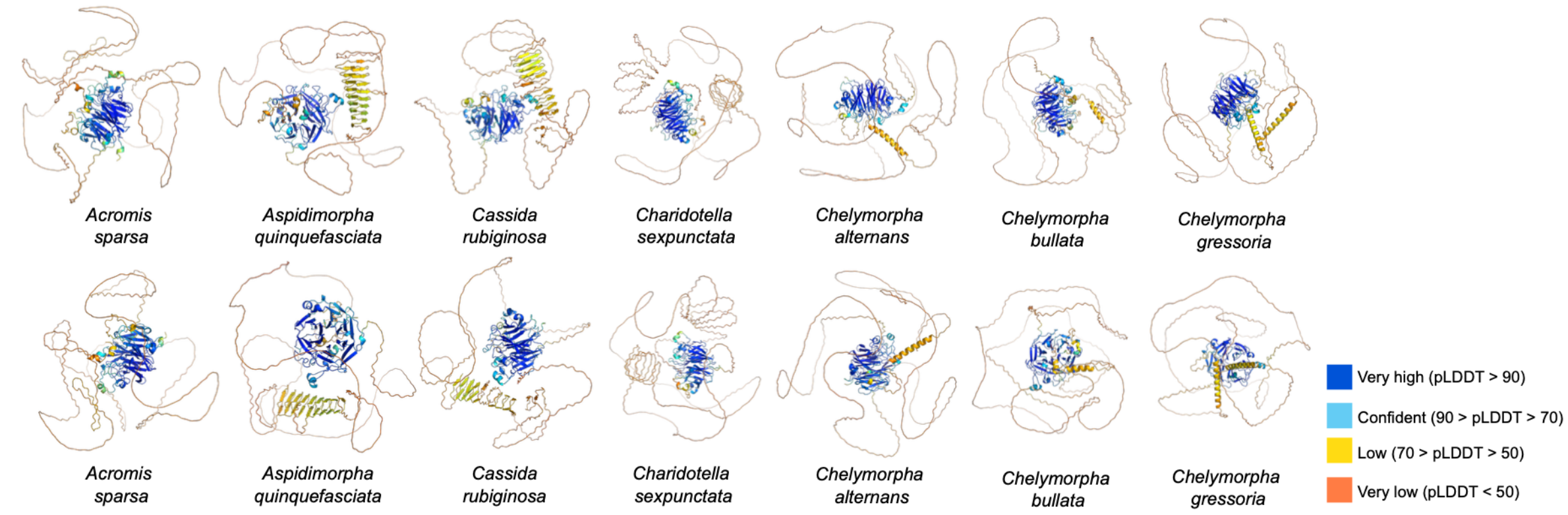

Figure S5. AlphaFold structural predictions of Yellow proteins from Cassidinae beetles, colored by confidence values. Predicted three-dimensional structures of Yellow proteins generated using AlphaFold. Models are colored according to per-residue prediction confidence (pLDDT score) and shown from two different orientations.

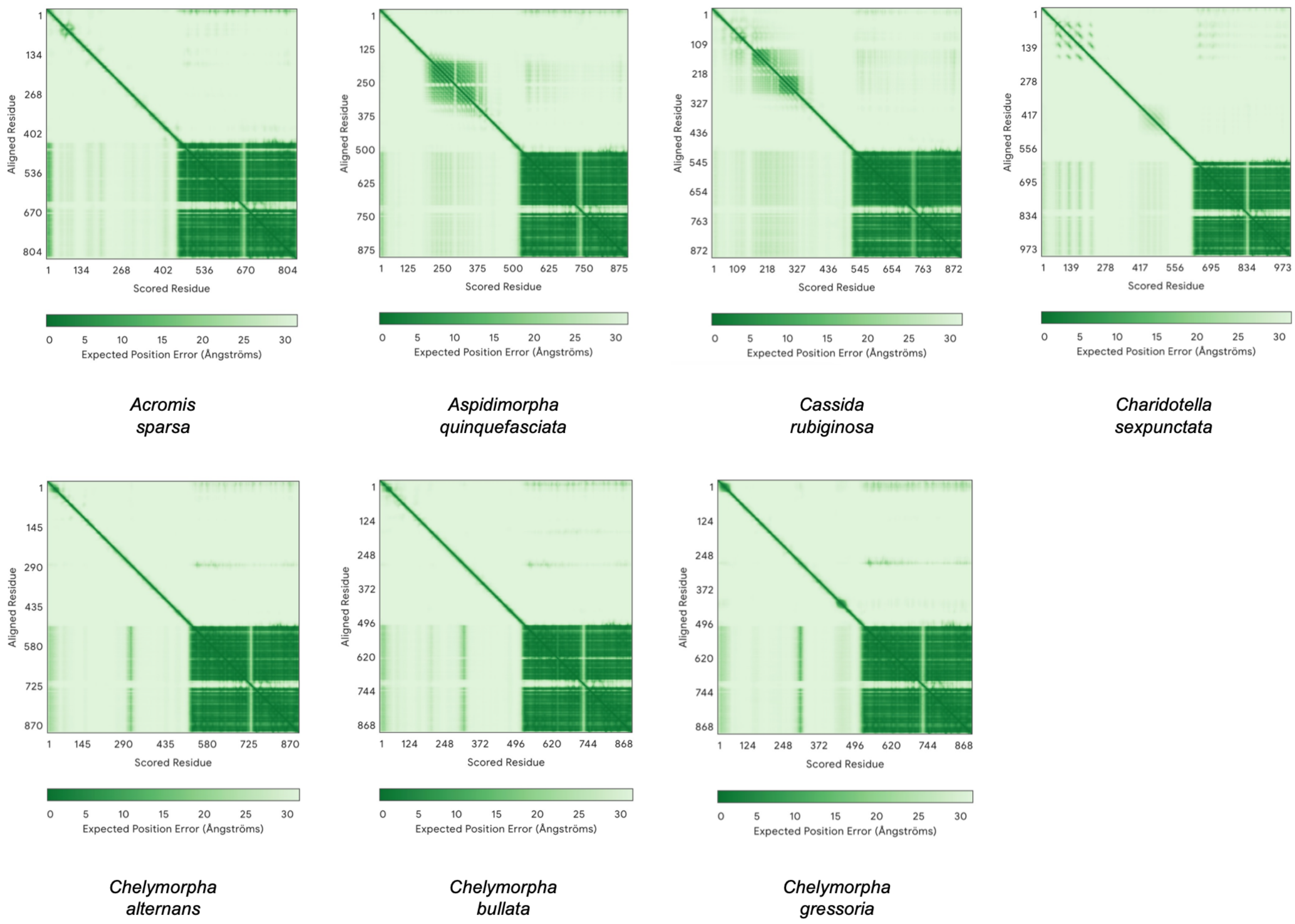

Figure S6. Predicted aligned error (PAE) plots for AlphaFold structural predictions of Yellow proteins from Cassidinae beetles. Related to Figure S5.

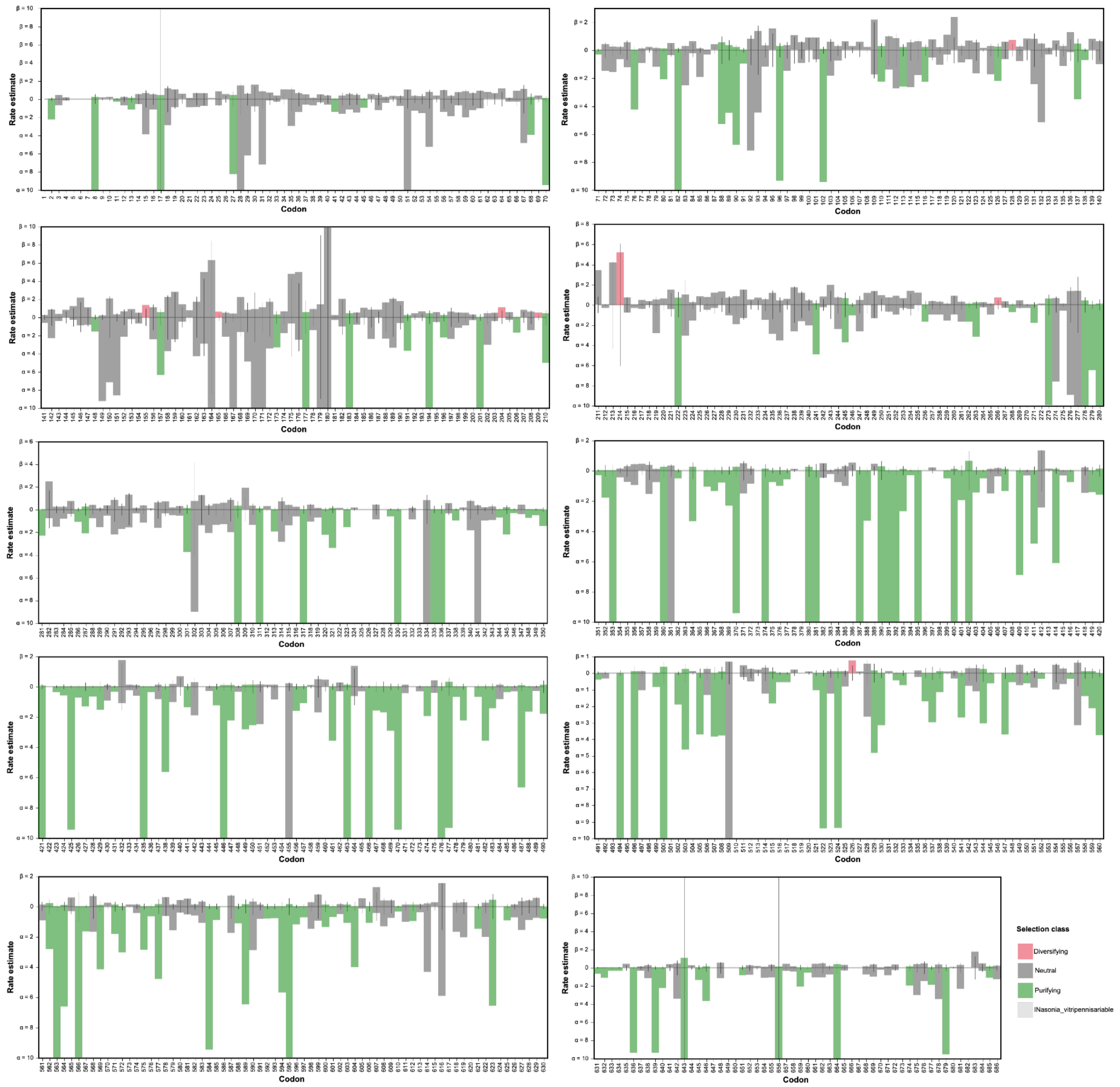

Figure S7. Results of FEL (Fixed Effects Likelihood) analysis showing maximum likelihood estimates of synonymous (α) and non-synonymous (β) substitution rates at each site, represented as bars. The line indicates the expected estimates under the null model (α = β). Estimates greater than 10 were censored at this value for visualization purposes. Sites with *p*-values < 0.1 were considered statistically significant. Codon sites correspond to the alignment of *yellow* genes from various Cassidinae species. Related to Figure 3.

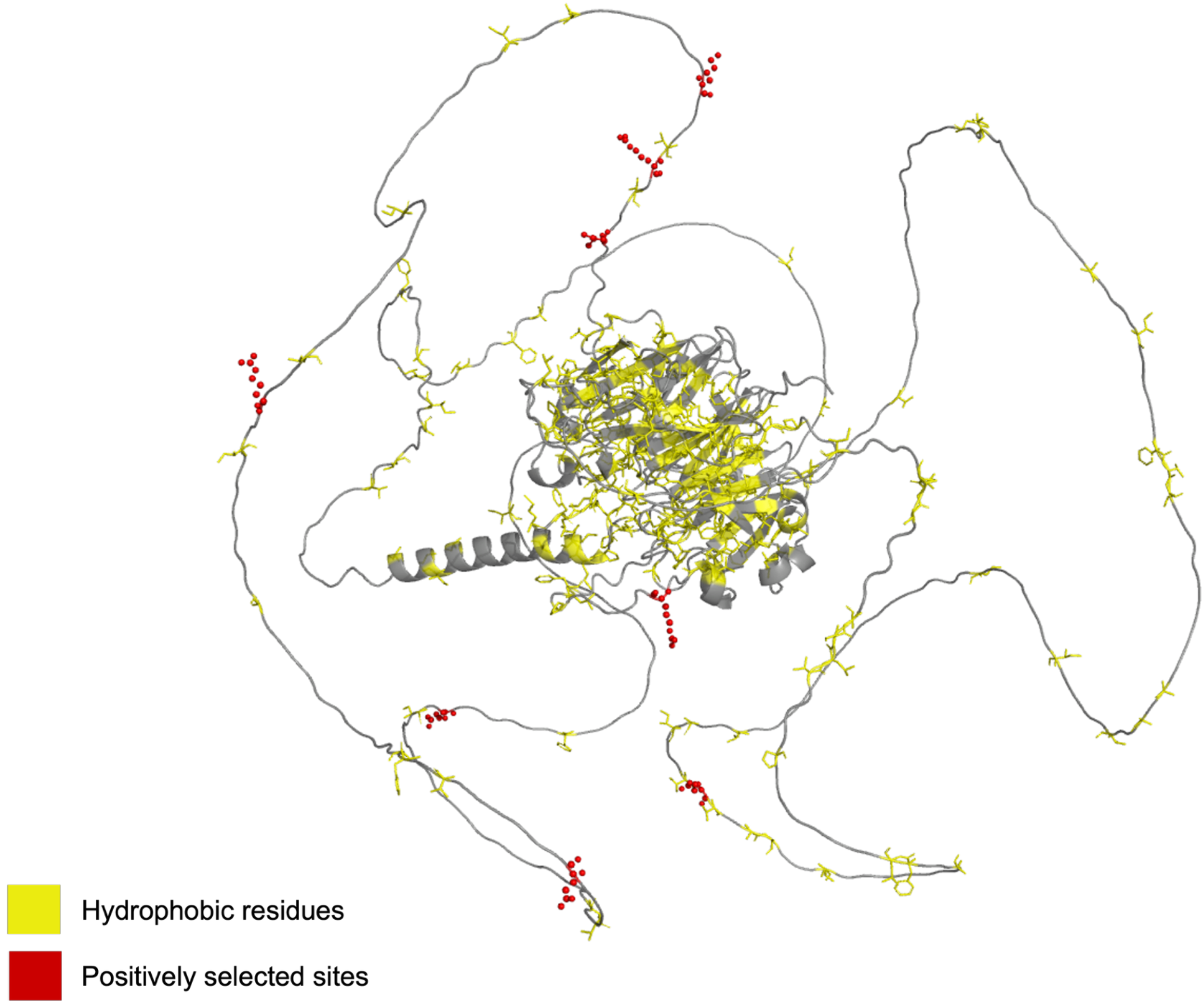

Figure S8. Structural mapping of hydrophobic residues and positively selected sites in *Chelymorpha alternans* Yellow protein. The predicted AlphaFold structure of the *C. alternans* Yellow is shown in grey cartoon and stick representation. Hydrophobic residues (Ala, Val, Leu, Ile, Met, Phe, Trp, and Pro) are highlighted in yellow stick format. Positively selected sites, identified through molecular evolution analysis, are shown in red spheres.

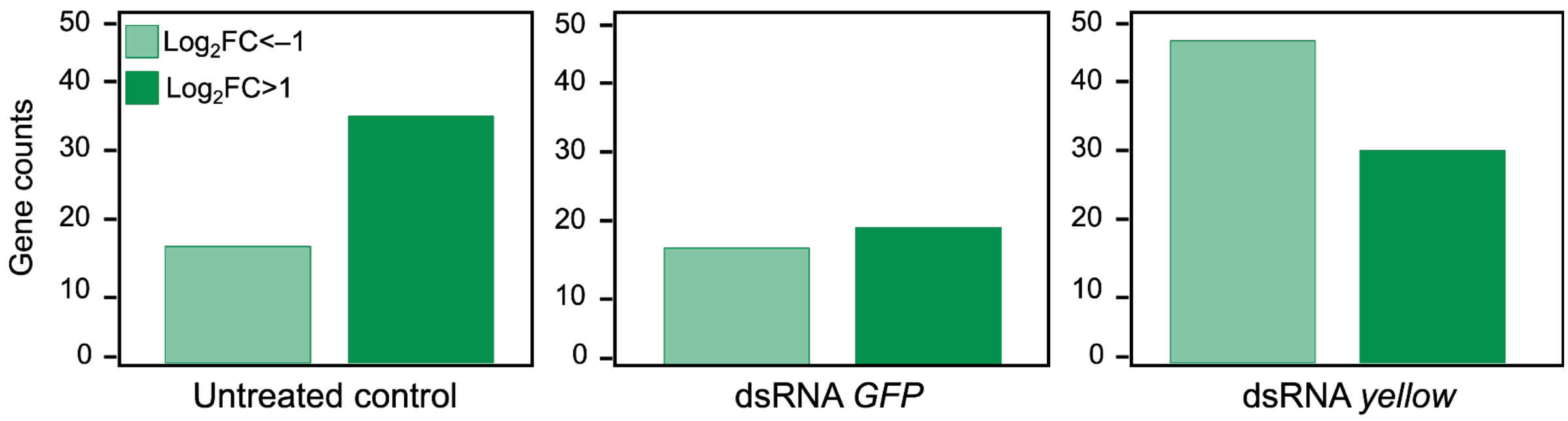

Figure S9. Number of genes with a $\log_2$ fold change (FC) < –1 or > 1 across different treatments under low-humidity conditions compared to control conditions obtained form DESeq2 analyses. A greater number of genes showed a $\log_2$FC < –1 in the dsRNA-*yellow* treatment compared to the untreated control and dsRNA-*GFP* treatments (Fisher's Exact Test, $p = 0.005199$). Related to Figure 7.

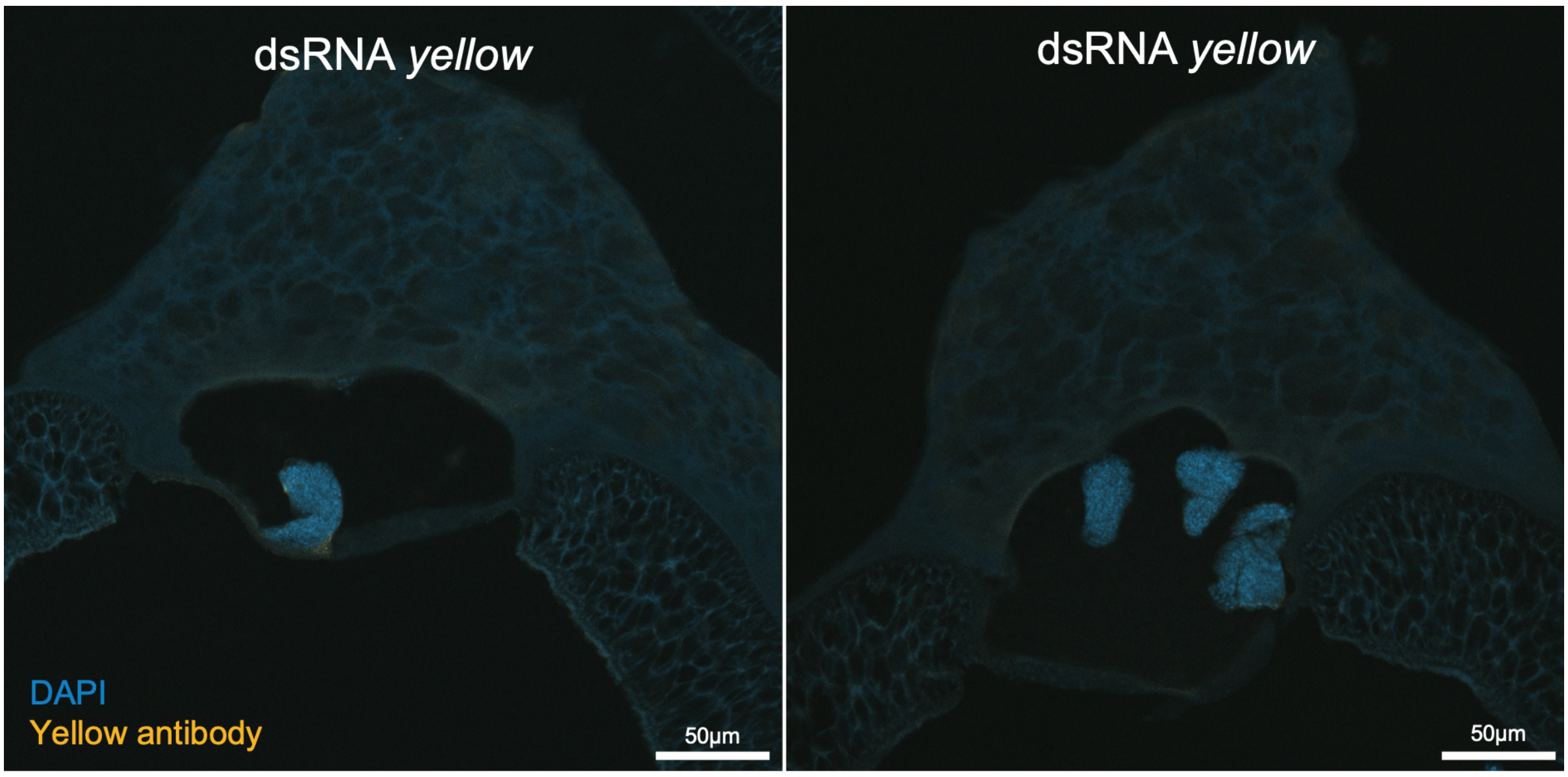

Figure S10. Independent replicate eggs from the dsRNA-yellow treatment showing Yellow protein localization. Cross-sections of eggs laid by females from the dsRNA-yellow treatment stained with an anti-Yellow antibody (yellow). DNA was stained with DAPI (blue). Images are from independent experiments replicating the results shown in Fig. 7B. Scale bars are shown for reference.

Table S1. Genome assembly and annotation statistics for *Chelymorpha alternans*. Related to Figure 1.

Genome size	No. Contigs	N50	GC content	BUSCO	Genes	mRNA	tRNA	Repeats (%)	Repeats (bp)
1.93 Gb	1031	9.7 Mb	33.17	98.2%	30425	26503	4996	68.80%	13345118

Table S2. Assembly statistics for de novo-assembled ovary-associated gland transcriptomes from various Cassidinae species.

Beetle species	Total assembled bases	Total trinity transcripts	Total trinity genes	N50	BUSCO (%)
Acromis sparsa	62669128	60352	42277	2141	89.5%
Aspidimorpha quinquefasciata	56145522	100441	83743	1094	78.7%
Cassida rubiginosa	57037515	53418	37834	2155	82.2%
Charidotella sexpunctata	75025493	66008	43167	2297	88.9%
Chelymorpha alternans	130936817	200819	148360	1091	81.1%
Chelymorpha bullata	69294026	57953	40906	2611	91.0%
Chelymorpha gressoria	45459678	41236	30910	2122	83.6%

Table S3. Candidate Yellow protein homologs identified by LC-MS from symbiont-bearing spheres of tortoise beetle species. The table shows the total number of peptides matched, sequence coverage, predicted molecular weight, protein sequence length, and the protein identification score. * Identification for *Charidotella sexpunctata* is tentative, the protein was detected with no unique peptide evidence, and the negative score indicates low confidence based on shared peptides only.

Species	Peptides	Sequence coverage [%]	Mol. weight [kDa]	Sequence length	Score
Aspidimorpha quinquefasciata	28	37.8	95.482	881	323.31
Chelymorpha gressoria	46	57.1	97.661	869	323.31
Cassida rubiginosa	13	16.4	93.792	866	202.32
Acromis sparsa	16	19.8	91.563	804	122.18
Charidotella sexpunctata	18	30.5	107.37	979	-2
Chelymorpha bullata	33	42.5	97.413	868	323.31

Table S4. AlphaFold pLDDT confidence scores for Yellow proteins in Cassidine beetles by exon region.

Species	Mean pLDDT	% pLDDT < 70	exon_1 mean pLDDT	exon_2_3 mean pLDDT
Acromis sparsa	53,61	59,00%	29,22	86,4
Aspidimorpha quinquefasciata	53,1	61,50%	32,01	85,96
Cassida rubiginosa	53,4	60,90%	31,94	86,59
Charidotella sexpunctata	46,65	65,90%	24,93	86,59
Chelymorpha alternans	51,12	60,70%	27,56	87,91
Chelymorpha bullata	51,18	61,30%	28,2	87,06
Chelymorpha gressoria	52,5	60,50%	29,64	88,24

Table S5. Gene-specific primers used to measure *yellow* and *EF-2* expression, and symbiont titers. Related to Figure 2, 4, 5, 6, and 7.

Gene	Product	Primer	Sequence (5'-3')	Orientation	Annealing temp (˚C)	Fragment size (bp)	Target	Reference
groL	60 kDa chaperonin	groL1_Chely_qpcr_F<br>groL1_Chely_qpcr_R	TGCTGCTTCTGTTGCTGGAT<br>TTCCACCCATTCCTGAACCA	Fwd<br>Rev	62	124	*Stammera* in *Chelymorpha alternans*	García-Lozano et al. (2024)
yellow (FUN_023368)	Major royal jelly protein/protein yellow	mrjp1_Chely_qpcr_F<br>mrjp1_Chely_qpcr_R	TGCAAGATCGAGTTCGGGTT<br>TGATCCTTGGGTTCCGTGAG	Fwd<br>Rev	60	171	*Chelymorpha alternans*	This study
EF-2 (FUN_008004)	Eukaryotic elongation factor 2	ef2_1_Chely_qpcr_F<br>ef2_1_Chely_qpcr_R	CATGGGACCCAATTATACGCCA<br>CCAAGAATTGATCGACACCGAC	Fwd<br>Rev	60	152	*Chelymorpha alternans*	This study

Table S6. Gene-specific primers used to synthesize double-stranded RNAs. Related to Figure 6 and 7.

Gene	Product	Primer	Sequence (5'-3')	Orientation	Annealing temp (˚C)	dsRNA length	Target	Reference
yellow	Major royal jelly protein/protein yellow	yellow_RNAi_F<br>yellow_RNAi_R	TATTCGGGGAACGATGGTAA<br>TTGAATGGCTAGCTTCGCTT	Fwd<br>Rev	60	688	*Chelymorpha alternans*	This study
GFP	Green fluorescent protein	GFP_RNAi_F<br>GFP_RNAi_R	TGGTGATGTTAATGGGCACAAA<br>AGCTCATCCATGCCATGTGTA	Fwd<br>Rev	63	651	pET30 plasmid	This study
