# Supplementary material for: Yellow protein co-opted to sustain obligate symbiosis in leaf beetles
Source: Nat Commun. 2026 Sep 3;17:9446. doi: 10.1038/s41467-026-76942-1 (PMC13538373; doi:10.1038/s41467-026-76942-1)
Supplement: Supplementary file 2 [file 41467_2026_76942_MOESM2_ESM.pdf]

Description of Additional Supplementary Files

Supplementary Data 1 - Differentially expressed genes in foregut symbiotic organs and ovary-associated glands.

Supplementary Data 2 - Site-by-site Fixed Effects Likelihood (FEL) analysis of yellow genes from Cassidinae species.

Supplementary Data 3 - Genes with $\log_2$ fold change (FC) in Stammera under control and low humidity conditions.

Supplementary Data 4 - Sequencing libraries generated in this study.

Supplementary Data 5 - Statistical analyses for Figures 2-7.
