# Supplementary material for: Yellow protein co-opted to sustain obligate symbiosis in leaf beetles
Source: Nat Commun. 2026 Sep 3;17:9446. doi: 10.1038/s41467-026-76942-1 (PMC13538373; doi:10.1038/s41467-026-76942-1)
Supplement: Supplementary file 3 [file 41467_2026_76942_MOESM8_ESM.pdf]

nature portfolio

Corresponding author(s): Hassan Salem

Last updated by author(s): 11/03/2026

Reporting Summary

Nature Portfolio wishes to improve the reproducibility of the work that we publish. This form provides structure for consistency and transparency in reporting. For further information on Nature Portfolio policies, see our Editorial Policies and the Editorial Policy Checklist.

Statistics

For all statistical analyses, confirm that the following items are present in the figure legend, table legend, main text, or Methods section.

n/a	Confirmed	
☐	☒	The exact sample size (*n*) for each experimental group/condition, given as a discrete number and unit of measurement
☐	☒	A statement on whether measurements were taken from distinct samples or whether the same sample was measured repeatedly
☐	☒	The statistical test(s) used AND whether they are one- or two-sided<br>*Only common tests should be described solely by name; describe more complex techniques in the Methods section.*
☐	☒	A description of all covariates tested
☐	☒	A description of any assumptions or corrections, such as tests of normality and adjustment for multiple comparisons
☐	☒	A full description of the statistical parameters including central tendency (e.g. means) or other basic estimates (e.g. regression coefficient) AND variation (e.g. standard deviation) or associated estimates of uncertainty (e.g. confidence intervals)
☐	☒	For null hypothesis testing, the test statistic (e.g. *F*, *t*, *r*) with confidence intervals, effect sizes, degrees of freedom and *P* value noted<br>*Give P values as exact values whenever suitable.*
☒	☐	For Bayesian analysis, information on the choice of priors and Markov chain Monte Carlo settings
☐	☒	For hierarchical and complex designs, identification of the appropriate level for tests and full reporting of outcomes
☒	☐	Estimates of effect sizes (e.g. Cohen's *d*, Pearson's *r*), indicating how they were calculated

Our web collection on statistics for biologists contains articles on many of the points above.

Software and code

Policy information about availability of computer code

Data collection

Proteomics data: Mascot and MaxQuant software
qPCR data: qPCRSoft software Analytik Jena

Data analysis

For beetle genome assembly HiFi reads (quality >Q20) were generated using the circular consensus sequencing (CCS) analysis via the pbccs tool from the pbbioconda package. CCS reads from both SMRT cells were merged and assembled into contigs using Hifiasm (v0.14.1-r314) with default parameters. The resulting contig-level assembly was filtered to remove heterozygous contigs using Redundans. Contaminant sequences were identified and removed with BlobTools2 (v1.1.1) and further deduplication was performed using the funannotate clean module from the Funannotate pipeline (v1.8.17). Genome assembly quality and completeness were assessed using BUSCO (v5.1.2). Genome annotation was conducted with the Funannotate pipeline (v1.8.17). Repetitive elements were identified and soft-masked via RepeatMasker (v4.0.7) and RepeatModeler (v2.0.3). Gene prediction combined ab initio models with transcriptomic evidence.

To study the transcriptional differences between foregut symbiotic organs and ovary-associated glands, raw reads were adapter-trimmed and quality-filtered with Trimmomatic (v0.36). Filtered reads were aligned to the Chelymorpha alternans reference genome using HISAT2 (v2.2.0). Gene-level counts were obtained with HTSeq (v0.11.5) (stranded=reverse -r pos) using the C. alternans genome annotation file. Differential gene expression was analyzed in R (v4.3.1) with the DESeq2 package, applying an adjusted False Discovery Rate (FDR) < 0.05 and a $\log_2$ fold-change > 2 as significance thresholds. A volcano plot illustrating differentially expressed genes was created with the EnhancedVolcano R package.

Phylogenetic analyses of Yellow proteins were performed by extracting the corresponding amino acid sequences in C. alternans alongside 269 Yellow amino acid sequences from hymenopteran, coleopteran, and dipteran insects, as well as six bacterial sequences. Sequences were aligned using MAFFT (v1.4.0) with default parameters. The best-fit substitution model was determined using ModelTest-NG (v0.2.0). A

maximum likelihood (ML) phylogenetic tree was constructed using RAxML-NG (v1.2.0) under the selected model.

To identify the Yellow proteins in additional tortoise beelte species, raw RNA seq reads were adapter-trimmed and quality-filtered using Trimmomatic (v0.36). De novo transcriptome assemblies were generated with Trinity (v2.8.5). Assembly completeness was evaluated with BUSCO (v5.1.2). Transcript abundance was quantified with RSEM. For each species, the top 10,000 transcripts were used to generate box plots of normalized transcript counts expressed as transcripts per million. Protein-coding sequences were predicted with TransDecoder (v5.5.0) and annotated with HMMER (v3.3.2) against the Pfam database. Sequences annotated as "Yellow" proteins were extracted from each species' predicted proteome and compared to the C. alternans Yellow sequence using BLAST.

Codon-level selection on the yellow gene was evaluated using the Fixed Effects Likelihood method implemented on the Datamonkey web server. Protein sequences from various tortoise beetle species were aligned with MUSCLE (v5.1), and a codon-aware nucleotide alignment was generated with PAL2NAL (v14). A maximum likelihood phylogeny was inferred automatically by Datamonkey and used alongside the codon alignment as input for the FEL analysis. Codon sites were considered to be under selection when the nonsynonymous substitution rate (dN) significantly differed from the synonymous rate (dS) at $p < 0.1$. The analysis identified 234 codon sites under purifying (negative) selection (dN < dS) and 8 sites under diversifying (positive) selection (dN > dS). To test for enrichment of positively selected sites within specific regions of yellow, the number of such sites in Exon 1 (7 sites) was compared to those in Exons 2 and 3 (1 site) using a Fisher's exact test. Exons 2 and 3 were grouped to represent the C-terminal region of the yellow gene. Region lengths were based on codon positions from the FEL alignment (346 codons in Exon 1; 340 codons in Exons 2 and 3). A second Fisher's exact test was applied to assess whether Exon 1 (69 sites) was significantly depleted of codon sites under purifying selection compared to Exons 2 and 3 (165 sites).

Candidate Yellow sequences were submitted to the AlphaFold3 server for structure prediction. Structural alignment of the predicted models was performed using the align function in PyMOL (v2.5.5) with C. alternans Yellow selected as the reference. A custom PyMOL (v2.5.5) script was used to calculate per-residue RMSD values by measuring distances between Cα atoms across the aligned models. RMSD values were stored in the B-factor field of the reference model and visualized using a red-blue color gradient, where red indicates low structural variability and low indicates high variability.
Model confidence scores (pLDDT) for each protein were extracted from AlphaFold predictions and summarized by exon region. Exon boundaries were defined based on the C. alternans Yellow gene model. For the remaining species, exon 1 and exons 2-3 were inferred by aligning transcript-derived sequences to the C. alternans reference due to the absence of annotated exon structures. To investigate putative functional sites, hydrophobic residues were identified in PyMOL (v2.5.5) and highlighted in yellow based on amino acid identity (Ala, Val, Leu, Ile, Met, Phe, Trp, and Pro).

For symbiont-Yellow cophylogenetic analysis, Yellow protein sequences from tortoise beetle species were aligned using MUSCLE (v5.1), including Leptinotarsa decemlineata (Coleoptera: Chrysomelidae: Chrysomelinae) as an outgroup. The optimal substitution model for the alignment was determined using ModelTest-NG (v0.2.0), For the symbiont phylogeny, 61 previously identified core symbiont protein sequences, along with sequences from four outgroup taxa (Buchnera aphidicola, Escherichia coli, Vibrio fisheri, and Candidatus Ishikawaella capsulata), were concatenated and aligned using MUSCLE (v5.1).
PartitionFinder (v2.1.1) was used to determine the best-fit substitution model for each partition with parameters set to branchlengths = unlinked, models = GTR, GTR+G, GTR+I+G, model_selection = bic. Maximum Likelihood (ML) phylogenies were inferred using RAxML-NG (v1.2.0) (ngen=1000) under the respective best-fit models.
The resulting trees were visualized side-by-side in Dendroscope3 as a tanglegram using the Neighbor Net Tanglegram algorithm. Cophylogenetic congruence between yellow genes and Stammera symbionts was assessed using eMPRess GUI (v1).

To study Stammera transcriptional response to low humidity stress in the absence of Yellow,
Raw RNAseq reads were adapter-trimmed and quality-filtered using Trimmomatic (v0.36). Filtered forward reads were aligned to the Stammera genome using Bowtie2 (v2.3.5.1). Gene counts were summarized with featureCounts 123 (-s 2) as part of the Subread package release 2.0.0. Differential gene expression was assessed independently within each treatment group (untreated control, dsRNA GFP and dsRNA yellow) across environmental conditions (control and low-humidity) using the DESeq2 package in R (v4.3.1), applying an adjusted False Discovery Rate (FDR) < 0.05 and a log2 fold change > 1 as significance thresholds.
To account for variability in bacterial biomass across samples, bacterial titers measured by qPCR were included as a covariate in the DESeq2 design formula (~ Bacterial_titer + Replicate + Treatment). Incorporating titer values into the differential expression model helped correct for global transcriptional shifts associated with differences in bacterial load, ensuring that observed changes in expression were attributed to dehydration responses, rather than variation in total bacterial RNA content. While DESeq2 accounts for library size differences through size factor normalization, this can lead to inflated fold-change estimates in samples with very low counts, including bacterial titers mitigated this issue. The number of genes with log2 fold-change < –1 or > 1 in untreated control and dsRNA GFP conditions were compared to those in the dsRNA yellow using a Fisher's exact test. Volcano plots were generated for each treatment group across environmental conditions using the EnhancedVolcano R package. Heatmaps of the log(x+1)-transformed normalized read counts were constructed using the pheatmap package to visualize expression patterns across samples.

To study the differences in yellow expression across female tissues, it was first confirmed that the data did not meet the normality assumptions using the Shapiro-Wilk test, data was futher analyzed using a Kruskal-Wallis test, followed by Wilcoxon rank-sum tests for pairwise comparisons. Bonferroni correction was applied to adjust for multiple testing.
Because yellow expression was undetectable in all male tissues, a Wilcoxon rank-sum test was used to assess sex-specific differences in expression. To assess age-related expression changes in the ovary-associated glands, a two-sample t-test was performed following confirmation of normality, comparing females 1 and 31 days following adult eclosion.
To analyze expression of yellow in segments of the ovary-associated glands, log transformation was applied to data from 1-day old females, while square root transformation was used for 31-day old females. After confirming assumptions of normality and homoscedasticity by Shapiro and Levene's tests, respectively, general linear models (LMs) were fitted separately for each dataset, with "segment" and "replicate" included as fixed factors. Post hoc pairwise comparisons between gland segments were conducted using Tukey's HSD test, implemented via the 'glht' function, with Bonferroni correction for multiple testing.
To compare yellow expression between ovary-associated glands and sphere-storing organs in 31-day old females, yellow/EF-2 ratios were averaged across gland segments to obtain a single expression value per organ. These were then normalized to the mean expression in the sphere-storing organs, yielding fold-change values. Data were log-transformed to meet normality assumptions, and differences between the two organs were assessed using a two-sample t-test.
To assess differences in symbiont titers across ovary-associated gland segments in 1- and 31-day old females. General linear models were fitted separately for each dataset with "segment" and "replicate" as fixed factors following Box-Cox transformation and subsequent confirmation of normality and homoscedasticity. Post hoc pairwise comparisons between segments were conducted using Tukey's HSD

implemented via the 'glht' function, with Bonferroni correction for multiple testing.
Stammera titers in sphere-storing organs of females 1 and 31 days following adult eclosion were also compared. Data were log-transformed and differences between timepoints were assessed using a two-sample t-test after validation of a normal distribution.
To study the effects of RNAi treatment in yellow expression, log transformed data were analyzed using general linear models (LMs) for each time point after verification that model assumptions were respected with "condition" and "replicate" included as fixed factors. Post hoc pairwise comparisons between treatment groups (untreated control, dsRNA GFP, and dsRNA yellow) were conducted using Tukey's HSD implemented via the 'glht' function, with Bonferroni correction for multiple testing.
To study the impact of yellow RNAi on symbiont titers under low-humidity conditions, statistical analyses were conducted separately for each collection day and treatment group. For eggs collected on day 9, symbiont copy numbers were modeled using a negative binomial generalized linear model (NB-GLM) with a log link function, implemented via the glm.nb() function in the MASS package. This model was chosen to account for overdispersion in the data, as evidenced by variance exceeding the mean, and was further supported by lower AIC values compared to alternative distributions.

For manuscripts utilizing custom algorithms or software that are central to the research but not yet described in published literature, software must be made available to editors and reviewers. We strongly encourage code deposition in a community repository (e.g. GitHub). See the Nature Portfolio guidelines for submitting code & software for further information.

Data

Policy information about availability of data

All manuscripts must include a data availability statement. This statement should provide the following information, where applicable:

- Accession codes, unique identifiers, or web links for publicly available datasets
- A description of any restrictions on data availability
- For clinical datasets or third party data, please ensure that the statement adheres to our policy

Genomic and transcriptomic sequencing data generated in this study have been deposited at the National Center for Biotechnology Information (NCBI) and are publicly available as of the date of publication under the BioProjects PRJNA1241163 and PRJNA1257053. SRA accession numbers for each dataset are indicated in Table S9. All code used in this study was written in bash, python and R programming language and scripts are available upon request.

Research involving human participants, their data, or biological material

Policy information about studies with human participants or human data. See also policy information about sex, gender (identity/presentation), and sexual orientation and race, ethnicity and racism.

Reporting on sex and gender	*Use the terms sex (biological attribute) and gender (shaped by social and cultural circumstances) carefully in order to avoid confusing both terms. Indicate if findings apply to only one sex or gender; describe whether sex and gender were considered in study design; whether sex and/or gender was determined based on self-reporting or assigned and methods used. Provide in the source data disaggregated sex and gender data, where this information has been collected, and if consent has been obtained for sharing of individual-level data; provide overall numbers in this Reporting Summary. Please state if this information has not been collected. Report sex- and gender-based analyses where performed, justify reasons for lack of sex- and gender-based analysis.*
Reporting on race, ethnicity, or other socially relevant groupings	*Please specify the socially constructed or socially relevant categorization variable(s) used in your manuscript and explain why they were used. Please note that such variables should not be used as proxies for other socially constructed/relevant variables (for example, race or ethnicity should not be used as a proxy for socioeconomic status). Provide clear definitions of the relevant terms used, how they were provided (by the participants/respondents, the researchers, or third parties), and the method(s) used to classify people into the different categories (e.g. self-report, census or administrative data, social media data, etc.) Please provide details about how you controlled for confounding variables in your analyses.*
Population characteristics	*Describe the covariate-relevant population characteristics of the human research participants (e.g. age, genotypic information, past and current diagnosis and treatment categories). If you filled out the behavioural & social sciences study design questions and have nothing to add here, write "See above."*
Recruitment	*Describe how participants were recruited. Outline any potential self-selection bias or other biases that may be present and how these are likely to impact results.*
Ethics oversight	*Identify the organization(s) that approved the study protocol.*

Note that full information on the approval of the study protocol must also be provided in the manuscript.

Field-specific reporting

Please select the one below that is the best fit for your research. If you are not sure, read the appropriate sections before making your selection.

☒ Life sciences ☐ Behavioural & social sciences ☐ Ecological, evolutionary & environmental sciences

For a reference copy of the document with all sections, see nature.com/documents/nr-reporting-summary-flat.pdf

Life sciences study design

All studies must disclose on these points even when the disclosure is negative.

Sample size	For each experiment, a sample size was chosen to obtain sufficient number of experiments and samples to calculate statistical significance. This was based on published studies, pre-experiments, and the difficulty to perform experiments. No statistical approach was performed to predetermine sample size.
Data exclusions	No collected data were excluded from the analysis.
Replication	Data presented were successfully replicated in at least 3 independent experimental groups.
Randomization	Allocation of individuals to cages or sampling was done randomly for each biological replicate.
Blinding	N/A

Reporting for specific materials, systems and methods

We require information from authors about some types of materials, experimental systems and methods used in many studies. Here, indicate whether each material, system or method listed is relevant to your study. If you are not sure if a list item applies to your research, read the appropriate section before selecting a response.

Materials & experimental systems

n/a	Involved in the study	
☐	☒	Antibodies
☒	☐	Eukaryotic cell lines
☒	☐	Palaeontology and archaeology
☐	☒	Animals and other organisms
☒	☐	Clinical data
☒	☐	Dual use research of concern
☐	☒	Plants

Methods

n/a	Involved in the study	
☒	☐	ChIP-seq
☒	☐	Flow cytometry
☒	☐	MRI-based neuroimaging

Antibodies

Antibodies used	custom-made polyclonal primary antibody (1:10 anti-Yellow) raised in rabbit (1.4 mg/ml, 0.02% sodium azide)<br>Alexa Fluor 568-conjugated goat anti-rabbit IgG (2 mg/ml; Thermo Fisher Scientific ; catalog number A-110011; lot number 2088068)<br>Alexa Fluor 488-conjugated anti-rabbit IgG secondary antibody (1:500) (Thermo Fisher Scientific; catalog number A-11008; lot number 6682-1)
Validation	anti-yellow validated in western blot analysis of symbiont spheres and purified protein and in immunochemistry analysis of paraffin-embedded tissue sections

Animals and other research organisms

Policy information about studies involving animals; ARRIVE guidelines recommended for reporting animal research, and Sex and Gender in Research

Laboratory animals	Tortoise beetle species (Coleoptera: Chrysomelidae: Cassidinae) including Chelymorpha alternans, Chelymorpha bullata, Chelymorpha gressoria and Aspidimorpha quinquefasciata were reared under controlled conditions at the Max Planck Institute for Biology (Tübingen, Germany): 26°C, 60% relative humidity, and a 14.5-hour light/9.5-hour dark cycle, with continuous access to their host plant, Ipomoea batatas. Experiments were performed at adult and egg stage for each species.
Wild animals	Charidotella sexpunctata and Acromis sparsa were collected in Gamboa, Panamá from leaves of Ipomoea batatas and Merremia umbellata, respectively. Cassida rubiginosa was collected from Cirsium oleraceum in Tübingen, Germany. Only between 1 to 3 adults were collected per species. Beetles were maintained at the Max Planck Institute for Biology (Tübingen, Germany): 26°C, 60% relative humidity, and a 14.5-hour light/9.5-hour dark cycle.
Reporting on sex	As the characterized protein is female-specific produced, sex was considered as an important factor in most of the experiments. To distinguish female and male adults we used the parameters described in:<br>C. R. Morrison, D. M. Windsor, The Life History of Chelymorpha alternans (Coleoptera: Chrysomelidae: Cassidinae) in Panamá. Ann. Entomol. Soc. Am. 111, 31–41 (2017).<br>"Female body length approximately 11–12 mm, width approximately 4–5 mm. Male body length is more variable, approximately 5–8 mm, width approximately 3–5 mm. Females are ovular with slight indentation at border of the pronotum and elytron. Males are more circular than females with pronounced dentition between the pronotum and elytron."

Field-collected samples	Beetles were maintained at the Max Planck Institute for Biology (Tübingen, Germany): 26°C, 60% relative humidity, and a 14.5-hour light/9.5-hour dark cycle. This study complies with Nagoya Protocol regulations in the Republic of Panama and France under the following collection and research permits: ARG-027-2023 and TREL2302365S/693.
Ethics oversight	Directive 2010/63/EU of the European Parliament and of the Council governs the protection of animals used for scientific purposes and applies to live non-human vertebrates (including independently feeding larvae and mammalian foetuses in the last third of development) and live cephalopods. It does not cover insects, which fall outside its scope. Nevertheless, our project adheres to the Three Rs principles to ensure ethical research practices: we apply Replacement by using insects instead of vertebrate animals. Reduction by carefully designing experiments to use only the minimum number of insects required for reliable results; and Refinement by maintaining high standards of care and handling to minimize stress and harm.

Note that full information on the approval of the study protocol must also be provided in the manuscript.

Plants

Seed stocks	Host plants for the different beetle species including Ipomea batatas, Merremia umbellata, and Cirsium oleraceum were used.
Novel plant genotypes	No novel plant genotypes were produced.
Authentication	The authentication of different species was carried out through phenotypic characterization of the grown plants.
