# Supplementary material for: Yellow protein co-opted to sustain obligate symbiosis in leaf beetles
Source: Nat Commun. 2026 Sep 3;17:9446. doi: 10.1038/s41467-026-76942-1 (PMC13538373; doi:10.1038/s41467-026-76942-1)
Supplement: Supplementary file 4 [file 41467_2026_76942_MOESM9_ESM.pdf]

nature portfolio

Peer Review File

Yellow protein co-opted to sustain obligate symbiosis in leaf beetles

Corresponding Author: Dr Hassan Salem

This file contains all reviewer reports in order by version, followed by all author rebuttals in order by version.

Version 0:

Reviewer comments:

Reviewer #1

(Remarks to the Author)
This paper found that yellow protein sustains endosymbionts in tortoise beetles. The spheres bearing the endosymbionts on caplets are loaded with Yellow, which protects the microbe. This is a tremendous finding for the science of insect-microbe interactions! I have no qualms about the science, and only minor quibbles about the presentation. Overall, this is an excellent and impactful submission!

The results are noteworthy, as we know so little about how endocellular symbionts are maintained. The extensive evidence and different tests described in this paper all support the findings. I find no flaws requiring major revision. The details are sufficient.

Please change the title from "beetles" to "tortoise beetles"

You need to be consistent with the italicization of Stammera. Comapre line 45 to the rest of the paper. Check what's the best way to do this in other papers, and I will trust the authors' judgement.

79-90 This should be introduction

Reviewer #2

(Remarks to the Author)
By integrating a combination of research approaches (including genomics, transcriptomics, RNAi, FISH, immunostaining, RT-qPCR, etc.), this study clarifies the critical mechanism underlying the role of the yellow gene in mediating the vertical transmission of the extracellular symbiont Stammera in tortoise beetles. In my opinion, the research follows a clear logical framework, employs appropriate methodologies, and yields highly reliable results. I have only a few minor revision suggestions as follows.
L 53: It is recommended that a brief introduction to the life history of Chelymorpha alternans be added around this section, such as how long it takes for female adults to become sexually mature and start laying eggs after emergence, and for how many consecutive days the females can sustain egg-laying. This is because the subsequent RNAi experiment mentions selecting female adults 31 days after emergence for injection, with sampling and detection conducted more than 30 days post-injection; this would inform readers that C. alternans has a relatively long lifespan and can possibly lay eggs continuously.
L 96-98: It is recommended to clearly specify that the female insects used for comparative transcriptome analysis are those in the oviposition period here.
L 99-100: “Among the most highly expressed genes in the ovary-associated glands was a member of the Yellow family….” It cannot be inferred from Figure 1J that the yellow gene has the highest expression level, should it be expressed as “the gene with the largest fold change of differential expression” here?
L148-154: again, here should be noted that sexually mature male and female beetles were used.
L158: “which begin 24 and 29 days after pupation”, should be after “eclosion”?
L205-206: From Figure 3B, how can we tell that the yellow gene ranks among the most highly expressed genes?
P14: the Figure 3C, the colors (black and gray) of the bootstrap support legend have low distinguishability; it is

recommended that they be replaced with a more distinct color scheme.
L610-611: should be “$|\log_2$ fold change$| > 2$”

Open Access This Peer Review File is licensed under a Creative Commons Attribution 4.0 International License, which permits use, sharing, adaptation, distribution and reproduction in any medium or format, as long as you give appropriate credit to the original author(s) and the source, provide a link to the Creative Commons license, and indicate if changes were made.
In cases where reviewers are anonymous, credit should be given to 'Anonymous Referee' and the source.
The images or other third party material in this Peer Review File are included in the article’s Creative Commons license, unless indicated otherwise in a credit line to the material. If material is not included in the article’s Creative Commons license and your intended use is not permitted by statutory regulation or exceeds the permitted use, you will need to obtain permission directly from the copyright holder.
To view a copy of this license, visit https://creativecommons.org/licenses/by/4.0/

REVIEWERS' COMMENTS

Reviewer #1 (Remarks to the Author):

This paper found that yellow protein sustains endosymbionts in tortoise beetles. The spheres bearing the endosymbionts on caplets are loaded with Yellow, which protects the microbe. This is a tremendous finding for the science of insect-microbe interactions! I have no qualms about the science, and only minor quibbles about the presentation. Overall, this is an excellent and impactful submission!

>>>> Thank you for the feedback and positive evaluation of our study.

The results are noteworthy, as we know so little about how endocellular symbionts are maintained. The extensive evidence and different tests described in this paper all support the findings. I find no flaws requiring major revision. The details are sufficient.

Please change the title from "beetles" to "tortoise beetles"
>>>> We have revised the title accordingly and replaced "beetles" with "leaf beetles" to more precisely reflect the taxonomic scope of the study.

You need to be consistent with the italicization of Stammera. Comapre line 45 to the rest of the paper. Check what's the best way to do this in other papers, and I will trust the authors' judgement.

>>>> Thank you for pointing this out. We have checked the formatting throughout the manuscript and revised it for consistency. According to the conventions for provisional bacterial taxa, *Stammera capleta* is italicized, whereas the status designation after "*Candidatus*" is not italicized. The formatting has been adjusted accordingly throughout the manuscript.

79-90 This should be introduction
>>>> We appreciate this suggestion. However, we believe that this information is better placed in the Results section, as it directly supports the interpretation of the first part of the results. Therefore, we have kept it in its original position to maintain the logical flow of the manuscript.

Reviewer #2 (Remarks to the Author):

By integrating a combination of research approaches (including genomics, transcriptomics, RNAi, FISH, immunostaining, RT-qPCR, etc.), this study clarifies the critical mechanism underlying the role of the yellow gene in mediating the vertical transmission of the extracellular symbiont Stammera in tortoise beetles. In my opinion, the research follows a clear logical framework, employs appropriate methodologies, and yields highly reliable results. I have only a few minor revision suggestions as follows.

>>>> We appreciate the feedback and positive evaluation of our study!

L 53: It is recommended that a brief introduction to the life history of Chelymorpha alternans be added around this section, such as how long it takes for female adults to become sexually mature and start laying eggs after emergence, and for how many consecutive days the females can sustain egg-laying. This is because the subsequent RNAi experiment mentions selecting female adults 31 days after emergence for injection, with sampling and detection conducted more than 30 days post-injection; this would inform readers that C. alternans has a relatively long lifespan and can possibly lay eggs continuously.

>>>> This is an important point, which we now clarify in line 93. Specifically, we note that oviposition commences 29 days after adult eclosion, which is why we chose to sample this developmental stage for transcriptomic sequencing.

L 96-98: It is recommended to clearly specify that the female insects used for comparative transcriptome analysis are those in the oviposition period here.

>>>> Thank you for the suggestion. We have clarified this point in the text. The sentence now reads: “To define the host traits involved in sphere production in tortoise beetles, we performed a comparative transcriptomic analysis of foregut symbiotic organs and ovary-associated glands in reproductively active *Chelymorpha alternans*.”

L 99-100: “Among the most highly expressed genes in the ovary-associated glands was a member of the Yellow family….” It cannot be inferred from Figure 1J that the yellow gene has the highest expression level, should it be expressed as “the gene with the largest fold change of differential expression” here?
>>>> We agree and have revised the sentence accordingly. It now reads: “Among the genes with the largest fold change in the ovary-associated glands was a member of the Yellow family, encoding a protein with a conserved MRJP domain (Figure 1J).”

L148-154: again, here should be noted that sexually mature male and female beetles were used.
>>>> We have clarified this point in the revised manuscript. The sentence now reads: “We investigated the expression dynamics of *yellow* across different tissue types and sexes in reproductively active *C. alternans* using reverse transcription-quantitative PCR (RT-qPCR).”

L158: “which begin 24 and 29 days after pupation”, should be after “eclosion”?
>>>> Thank you for pointing this out. We have corrected “pupation” to “eclosion” in the revised manuscript.

L205-206: From Figure 3B, how can we tell that the yellow gene ranks among the most highly expressed genes?

>>>> We have revised the sentence to clarify the comparison. It now reads: Similar to *C. alternans* (Figures 1 and 2), a *yellow* homolog ranked among the most highly expressed genes compared to the median transcript expression within the transmission organs across all species (Figure 3B).

P14: the Figure 3C, the colors (black and gray) of the bootstrap support legend have low distinguishability; it is recommended that they be replaced with a more distinct color scheme.

>>>> We have revised the figure and replaced the color scheme with more clearly distinguishable colors.

L610-611: should be “$|\log_2$ fold change$| > 2$”

>>>> Corrected accordingly in the revised manuscript.
